# Investigation of Chemical and Processing Hurdle Impacts upon Microbial Control and Physicochemical Quality of Manufactured Pepperoni Products

**DOI:** 10.3390/foods15111952

**Published:** 2026-06-01

**Authors:** Ciarán H. Crowley, Joseph P. Kerry, Geraldine Duffy

**Affiliations:** 1 School of Food and Nutritional Sciences, University College Cork, College Road, T12 T997 Cork, Ireland; 116402292@umail.ucc.ie; 2 Teagasc Ashtown Research Centre, Ashtown, D15 DY05 Dublin, Ireland

**Keywords:** hurdle technology, fermented foods, reformulation, salt reduction, nitrite reduction, dry-fermented meats, pepperoni

## Abstract

This study investigated the stage-dependent effects of salt, nitrite, heat treatment, and water activity (aW) on physicochemical and microbiological parameters during fermented meat manufacture. Eight formulations representing a fractional subset of a 2 × 2 × 3 × 2 factorial design were evaluated across four processing stages (pre-fermentation, post-fermentation, post-heat treatment, and post-drying). A stage-resolved multivariate analysis of variance (MANOVA) framework was applied, followed by univariate testing with Benjamini–Hochberg false discovery rate (FDR) correction. Results demonstrated that hurdle effects were strongly stage dependent. Nitrite and its interaction with salt were associated with early stage microbial variation (η^2^ ≤ 0.72), while heat treatment was the dominant factor influencing microbial reduction at the post-processing stage. Notably, short high-temperature heat treatments were most effective at microbial reduction, followed by longer lower-temperature heat treatments, with the intermediate treatment being the least effective, indicating that equivalent time–temperature combinations did not yield equivalent microbiological outcomes. At the final stage, salt and nitrite were associated with compositional, microbial, and quality-related parameters, while aW influenced product structure and concentration effects but was not retained as an independent factor in the multivariate model. These findings indicate that hurdle effects evolve throughout processing and should be considered within a dynamic, stage-specific framework. The results provide a basis for the development of integrated reformulation strategies to reduce salt and nitrite levels while maintaining microbial stability and product quality.

## 1. Introduction

Salt and nitrite are cornerstone ingredients in the manufacture of fermented meat products, where they perform multiple technological, biochemical, and microbiological functions essential to product safety, stability, and quality [1]. Sodium chloride contributes to curing through protein solubilisation and water binding, facilitates flavour development, and plays a critical role in reducing water activity (aW), thereby limiting the growth of pathogenic and spoilage microorganisms [2]. Nitrite, typically added as sodium nitrite or generated from nitrate through microbial reduction, is central to cured colour development via the formation of nitrosylmyoglobin, contributing to the characteristic flavour, delaying lipid oxidation, and exerting a strong inhibitory effect against key pathogens, particularly Clostridium botulinum [3,4].

Beyond their direct antimicrobial effects, salt and nitrite influence the biochemical environment of fermented meats by modulating enzyme activity, fermentation kinetics, and microbial ecology [5]. Salt affects the rate and extent of acidification by lactic acid bacteria (LAB), while nitrite interacts with both endogenous muscle enzymes and starter culture metabolism [3,6,7]. Collectively, these effects contribute to product stability during fermentation, drying, and storage, ensuring consistency, shelf-life, and consumer acceptability [2,8].

Despite their technological importance, the reduction in salt and nitrite in fermented meat products, which has always been controversial in terms of cured meat manufacture, has become more so in recent years owing to constant criticism and highlighted health concerns raised by various health and regulatory groups, particularly in relation to salt. Excess dietary sodium intake is strongly associated with hypertension and elevated cardiovascular disease risk, prompting international health authorities to advocate for population-wide sodium reduction strategies [9]. The World Health Organization recommends limiting sodium intake to less than 2 g per day (equivalent to approximately 5 g salt per day) in adults as a means of reducing blood pressure and cardiovascular disease risk [10]. Within the European Union, this has translated into coordinated reformulation efforts, including a common framework for voluntary national salt reduction initiatives that established a benchmark of at least 16% salt reduction over four years (baseline ~2008) across major food categories, including processed and meat-based products [11]. While salt remains functionally indispensable in fermented meat systems, particularly for water activity control, microbial inhibition, and sensory quality, there is increasing incentive to minimise its use without compromising safety, quality and consumer appeal [2].

Nitrite has also been the subject of sustained scientific and regulatory evaluation due to its role in the formation of N-nitroso compounds under nitrosating conditions [12]. Nitrite contributes directly to cured colour development, oxidative stability, and inhibition of Clostridium botulinum [13,14]. However, it can also participate in endogenous nitrosation reactions, leading to the formation of nitrosamines, several of which have demonstrated carcinogenicity in animal models [12]. The International Agency for Research on Cancer (IARC) has classified processed meat as carcinogenic to humans (Group 1), identifying curing and preservation processes as contributors to the formation of carcinogenic compounds, including N-nitroso compounds [15]. Additionally, IARC has concluded that ingested nitrate or nitrite under conditions that result in endogenous nitrosation is probably carcinogenic to humans (Group 2A) [16]. While not definitive in any way, these evaluations have played a key role in shaping both consumer perception and regulatory policy surrounding cured meat products [17].

At the regulatory level, the use of nitrite and nitrate as food additives is tightly controlled within the European Union under Regulation (EC) No 1333/2008 [18]. For non-heat-treated meat products, including fermented sausage-type products such as pepperoni, the maximum permitted amounts that may be added are 150 mg/kg for sodium nitrite (E 250) and 150 mg/kg for sodium nitrate (E 251), respectively. These limits reflect a risk-management approach that balances technological necessity with toxicological considerations. Complementing this legislative framework, international risk assessments by bodies such as the European Food Safety Authority and the Joint FAO/WHO Expert Committee on Food Additives have established acceptable daily intakes of 0–0.07 mg/kg body weight/day for nitrite and 0–3.7 mg/kg body weight/day for nitrate, with nitrosamine formation recognised as a critical factor in overall risk evaluation [19]. Together, these public health recommendations and regulatory constraints provide strong motivation for the development of reformulation strategies capable of reducing salt and nitrite levels in fermented meat products while maintaining safety, quality, and technological functionality.

One promising strategy for enabling salt and nitrite reduction in fermented meat products is the application of hurdle technology. Hurdle technology is based on the deliberate combination of multiple preservation factors; each applied at sub-lethal intensity, to collectively inhibit spoilage microorganisms while maintaining product quality [20]. Rather than relying on a single dominant preservative, this approach exploits synergistic interactions between physical, chemical, and biological hurdles, thereby enhancing microbial stability while theoretically allowing for reduced levels of individual additives such as salt and nitrite [21,22]. In fermented meat systems, microbial safety and stability are traditionally achieved through the combined effects of salt-induced water activity reduction, nitrite-mediated inhibition, fermentation-driven acidification, and competitive starter culture activity [23,24]. Reducing salt and nitrite disrupts this established balance, potentially increasing susceptibility to spoilage species survival or outgrowth. Hurdle technology provides a framework through which alternative or compensatory barriers can be strategically introduced to maintain microbial control under reformulated conditions [7,25].

Heat-treatment represents a particularly effective physical hurdle when integrated into fermented meat processing. Controlled thermal exposure can impose direct lethality or sub-lethal injury on spoilage microorganisms, thereby reducing viable populations and impairing stress response mechanisms. Even when applied at temperatures insufficient to fully sterilise the product, heat treatment can significantly enhance overall microbial control by increasing cellular sensitivity to other hurdles such as low pH and subsequent reduction in water activity [26,27]. Importantly, when applied after fermentation, heat treatment can be designed to preserve desirable fermentation-derived attributes while selectively targeting less resilient microbial populations [28].

Water activity reduction constitutes a complementary and essential hurdle in fermented sausage manufacture. Lowering aW through controlled drying restricts microbial metabolism, enzyme activity, and cellular replication, particularly in salt- and nitrite-sensitive organisms [29]. Reduced water availability also amplifies the effectiveness of other hurdles by limiting microbial repair mechanisms following stress exposure. In reformulated systems with reduced salt content, achieving sufficient aW reduction becomes especially critical to compensate for the loss of salt’s osmotic and antimicrobial effects [30].

When combined, heat treatment and water activity reduction exert synergistic effects that extend beyond their individual contributions. Heat-induced cellular injury can increase susceptibility to osmotic stress, while low aW environments can suppress recovery and regrowth following thermal exposure [31]. These interactions are further reinforced by fermentation-associated acidification and the activity of starter cultures, which contribute to competitive exclusion, rapid pH decline, and metabolic by-products inhibitory to undesirable microorganisms [32]. Starter cultures selected for robustness under thermal and low-moisture conditions can therefore play a pivotal role in stabilising reformulated systems subjected to multiple hurdles [33].

Within this context, the present study aimed to evaluate the independent and combined effects of key processing hurdles—salt, nitrite, heat treatment, and water activity—on physicochemical and microbial parameters throughout the pepperoni manufacturing process. An established, commercially produced pepperoni formulation was used as a model system, from which a series of modified recipes was generated to systematically vary these factors across four processing stages: pre-fermentation, post-fermentation, post-heat treatment, and post-drying.

A central feature of this work is the application of a stage-resolved multivariate analysis of variance (MANOVA) framework, enabling quantification of hurdle effects within and across discrete processing stages. This approach allows for assessment of both independent and interacting influences of processing factors within a temporally structured system, providing a more detailed understanding of how hurdle effectiveness evolves throughout manufacture. Using this framework, the study examines whether: (i) reduction in salt and/or nitrite is feasible in the context of overall product safety and quality, and (ii) the strategic integration of heat treatment and water activity reduction can provide a stable and complementary approach to maintaining microbial and physicochemical integrity in reduced-salt and reduced-nitrite formulations.

While previous studies have examined the influence of processing parameters in fermented meat systems (e.g., Fraqueza et al., 2021) [7], the present work extends this by applying a stage-specific, multivariate statistical approach to characterise the evolving contributions of multiple hurdles throughout processing. This framework aligns with contemporary food safety and reformulation objectives by identifying stage-dependent control points and informing integrated strategies for product optimisation.

## 2. Materials and Methods

### 2.1. Experimental Design

This study employed a multifactorial experimental design to evaluate the stage-dependent effects of salt level, nitrite level, heat treatment, and water activity during pepperoni manufacture, a popular fermented meat product. Eight pepperoni formulations were produced under controlled pilot-scale conditions, by: varying salt concentration (2.5% vs. 1.4%), nitrite concentration (150 ppm vs. 50 ppm), heat treatment (three temperature–time profiles: HT1—53.5 °C for 60 min; HT2—61 °C for 40 min; and HT3—64 °C for 20 min), and target water activity (0.91 vs. 0.94), as presented in Table 1. This design represented a fractional subset of a nominal 2 × 2 × 3 × 2 factorial structure (salt level × nitrite level × heat treatment × water activity). This approach was adopted to ensure practical feasibility while capturing key contrasts between processing conditions. As a result, not all main effects and interactions were fully orthogonal, and certain factor combinations, particularly involving heat treatment, were not independently estimable across all formulations. Accordingly, results were interpreted within the constraints of the observed design space. All other formulation components and processing conditions were standardised across treatments. Products were manufactured as described in Section 2.2, and analysis was conducted following the protocol described in Section 2.3.1. Each formulation was produced in three independent sample batches (three distinctly different manufacturing occasions), which represented the primary biological replicates within the study design. Replicate analytical measurements were performed within each batch to capture analytical variability and improve measurement reliability. The design enabled evaluation of both independent formulation effects and selected interactions across processing stages, with particular emphasis placed on salt–nitrite interactions and stage-dependent hurdle behaviour during pepperoni manufacture.

### 2.2. Manufacture

All ingredients for the manufacture of pepperoni recipes were obtained from Dawn Farms Foods (Naas, Kildare, Ireland). The recipe ingredients were as follows; Pork shank 32%, Pork trim 32%, Beef fat 31%, Dry spice 3% (a confidential industrially defined combination of onion powder, spices and dextrose), Free salt (1.4–2.5% as per recipe), Sodium Nitrite (0.015–0.005% as per recipe), Paprika Oleoresin 0.02%, Rosemary oleoresin 0.13%, Liquid spice 0.29% and LAB culture (Christian Hannsen, Hørsholm, Denmark) 0.03%.

Upon receipt of the raw ingredients, these were divided into batches so that (n = 18) 250 g pepperoni would be produced per batch. Meat ingredients (Pork shank 32%, Pork trim 32%, Beef fat 31%) were weighed into polyamide/polyethylene vacuum bags (Alcom, Campogalliano, Italy), vacuum packed using a Webomatic Vacuum packaging system (Werner Bonk, type D463, Bochum, Germany) and stored at −18 °C until manufacture. Liquid spices and dry ingredients were pipetted/weighed into 10–100 mL Rohr tubes (Sigma-Aldrich, Arklow, Ireland) and stored at 3 °C until manufacture. LAB culture (HPS) (Christian-Hansen, Hørsholm, Denmark) was stored at −80 °C, weighed and diluted in 2 mL dH20 on the day of manufacture.

Pork shank and trims were partially defrosted overnight at 3 °C, while beef backfat was not defrosted prior to manufacture. Meat ingredients were loaded into a clean, dry PVC tray; frozen chunks were broken partially and distributed across the tray. The meat was minced via a 2 mm pore or plate size, fully mincing and blending the meat ingredients. The minced ingredients were dispersed uniformly across the base of the tray prior to the uniform distribution of all dry ingredients and oleoresins. All ingredients were thoroughly kneaded into the meat mixture by hand until the myofibrillar structure of the meat had become visually dissociated, all ingredients were homogenously incorporated, and the texture resembled a batter. Finally, the solubilized LAB culture was distributed evenly throughout the batter and further hand-mixed until uniformly dispersed. Upon the complete homogenous incorporation of all ingredients, the batter was extruded or stuffed into 45 mm cellulose casings (Walsroder, Walsrode, Germany), which were previously tempered in tepid distilled water prior to usage. Each pepperoni sausage (approx. 250 g) was weighed and labelled prior to fermentation. The pepperoni was fermented in a Ness fermentation chamber/smoker/oven (GERMOS NESS GmbH & Co. KG©, Remshalden, Germany). Temperature probes were inserted in two pepperoni sausage samples from each batch, which monitored core temperature from the beginning of fermentation to the end of heat-treatment following application. Fermentation occurred at 37 °C and a relative humidity (RH) of 85% for approximately 8 h., until the pH had decreased from the initial value of ~5.65 to <5.1. Heat-treatment was initiated at the fermentation endpoint. Prior to temperature elevation to the set heat-treatment, the pepperoni sausages were tempered with an intermediate step (to prevent heat-shock induced fat liquidation), to a core temperature of 47 °C for 20 min. Subsequently, the respective heat treatment was implemented. Upon completion of the heat treatment, the pepperoni was showered with cold water for 20 min, rapidly terminating the cooking process. The pepperoni samples were briefly removed from the chamber, which was cleaned and dried, prior to their reinsertion for the drying stage. The chamber was set to 15 °C with continuous airflow and a relative humidity of 65%, in which the pepperoni dried to their respective target aW (±0.01) within 7–9 days. Throughout the manufacturing process pH and aW were continuously monitored. Each pepperoni recipe was manufactured in three independent production batches or three independent commercial trials, to which the same testing protocol was applied.

### 2.3. Sampling and Testing Procedure

#### 2.3.1. Protocol of Analysis

Physicochemical and microbial analysis were conducted at four stages during the manufacturing process, namely: pre-fermentation, post-fermentation, post-heat treatment and at the endpoint of drying. Proximal compositional analysis (moisture, fat, protein, ash) was carried out at each of the four stages (protein was not assessed for post-heat treatment). Physicochemical tests (aW and pH) were assessed at each of the four stages. Salt, nitrate, nitrite and TBARS were assessed at pre-fermentation, post-fermentation and at the dry stage. Lactic acid percentage, colour (L*, a*, b*) and texture profile analysis (TPA) were assessed at the dry stage only. Microbial quantification of all species was assessed at each of the four stages of manufacture.

At each stage of manufacture, four pepperoni sausage samples were removed from each batch for analysis of the respective physicochemical and microbiological parameters. Two pepperoni samples were maintained in their whole state for duplicate microbial analysis and subsequently used for TPA and Colour (L* a* b*) analysis (at the dry stage only). Two pepperoni samples were homogenised and used for all physicochemical analyses.

#### 2.3.2. Preparation of Pepperoni Homogenate

Upon completion of drying, casings were removed from the samples, the pepperoni was diced into large chunks (2 cm × 2 cm) and placed into a Büchi laboratory blender (BÜCHI Labortechnik AG, Flawil, Switzerland), and homogenised. Homogenates were weighed in 50 g portions and placed into polyamide/polyethylene laminate pouches, vacuum-packed and stored at −20 °C until analysis.

#### 2.3.3. Preparation of 1:10 Homogenate Dilutions

Homogenates were diluted in distilled water to create a 1:10 dilution. Pepperoni homogenate (50 g) was weighed into a 500 mL Duran bottle (DURAN^®^, DWK Life Sciences Ltd., Stoke-on-Trent, UK). Homogenates were suspended in 450 mL of distilled water. The bottle was sealed and shaken vigorously by hand for 2 min. These samples were then filtered through Whatman filter paper #1 (Cytiva, Carrigaline, Ireland) into 500 mL glass beakers, in which the clear filtrate was prepared for analysis of NaCl content, pH and total titratable lactic acid.

### 2.4. Analytical Methods

#### 2.4.1. Physicochemical Methods

Proximal Composition (Moisture, Fat, Protein and Ash)

Moisture and fat contents were determined using a CEM SMART Trac II analyzer (CEM Corporation, Matthews, NC, USA). For each measurement, 2–3 g of homogenised sample was weighed on the instrument balance, spread evenly across a CEM paper sheet, and covered with a second sheet. The prepared sample was loaded into the analyzer, where moisture was determined by microwave drying. Following moisture analysis, the sample was transferred to a CEM film envelope, compressed into a Fast Trac II tube, and subjected to NMR-based fat analysis. Protein content was determined by the Kjeldahl method in accordance with AOAC Official Method 981.10 [34]. Approximately 0.5 g (±0.1) of accurately weighed pepperoni homogenate was used per analysis. Duplicate analysis was conducted on each of the isolated homogenates.

The ash content of pepperoni samples was determined using a muffle furnace in accordance with the AOAC Official method 942.05 [35]. Approximately 5 g of each homogenised sample was accurately weighed into a pre-weighed, dry crucible. The crucibles were placed in a muffle furnace and incinerated at 550 °C for 6 h. to ensure complete combustion of organic matter. After incineration, the crucibles were allowed to cool at room temperature in a desiccator to prevent moisture absorption. The final weight of the ash was recorded, and the percentage ash content was calculated using the following formula.

$$Ash\;content\%=\left(\frac{ash\;weight(g)}{sample\;weight(g)}\right)\times 100$$

pH, Salt and Lactic acid (%)

Extracted homogenates from the four manufacturing testing stages were quantified for pH via the Mettler Toledo FiveEasy pH Meter (Mettler Toledo, Columbus, OH, USA), by which the pH of the (1:10) pepperoni homogenate solutions were quantified, the latter being presented in this study. Triplicate measurements were conducted on each of the homogenates isolated per recipe, per stage.

Salt (NaCl) was quantified via an adaptation of the Mohr method [36], adapted by [37]. A 10 mL aliquot of the filtrate was transferred into a clean beaker, and a few drops of potassium chromate (K_2_CrO_4_) indicator were added. The sample was titrated with standardised 0.1 mol L^−1^ silver nitrate (AgNO_3_) until a reddish-brown endpoint was observed, indicating the formation of silver chromate (Ag_2_CrO_4_). The volume of AgNO_3_ used was recorded, and the NaCl content was calculated using the formula:

$$NaCl\%=\left(\frac{V\times N\times 58.44}{W}\right)\times 100$$

where

V = Volume of AgNO_3_ (mL)

N = The normality of AgNO_3_,

58.44 = Molecular weight of NaCl,

W = Weight of the sample (g).

Total titratable lactic acid (TTLA) content was determined by titration of the sample with 0.1 M NaOH as an adaptation of the method for the quantification in cheese [38] as described by [39]. A 25 mL aliquot of the previously prepared 1:10 homogenate dilution was transferred into a clean beaker, and three drops of phenolphthalein indicator solution (1% *w*/*v* in ethanol) were added. The sample was titrated with standardised 0.1 N NaOH while being continuously stirred until a persistent light pink colour was observed, indicating equilibrium. The titration volume was recorded and used to determine lactic acid content using the formula below:

$$Lactic\;acid\%=\left(\frac{V\times N\times 90.08}{W}\right)\times 100$$

where

V = is the volume (mL) of NaOH used,

N = is the normality of NaOH,

90.08 = Molecular weight of lactic acid,

W = Sample weight (g).

aW

Water activity (aW) of the pepperoni samples was measured using an AQUALAB 4TE water activity meter (Meter Group, Pullman, WA, USA). Triplicate measurements were conducted on each of the homogenates isolated per recipe, per stage.

Colour

Instrumental colour assessment was conducted using a Minolta CR- 300 colorimeter (Konica Minolta, Osaka, Japan). The colour coordinates assessed included lightness (L*), redness (a*), and yellowness (b*). Triplicate measurements were conducted on each of the homogenates isolated per recipe, per stage.

Nitrate and Nitrite

The nitrate content of pepperoni samples was determined following the AOAC Official Method 993.03 [40], with modifications to the reduction stage for health and safety. Nitrite determination of samples was carried out in accordance with AOAC Official Method 973.31 [41]. A total of 5 g of sample was used per sample analysis. Duplicate analysis was conducted on each homogenate isolated.

TBARS

To quantify lipid oxidation, Thiobarbituric Acid-Reactive Substances (TBARS) were assessed as described by [42]. Pepperoni homogenate (2.5 ± 0.1 g) was accurately weighed per analysis. The sample was transferred to a 50 mL screw-capped Rohr tube (Sarstedt, Germany) and 20 mL of distilled water and 10 mL of 25% trichloroacetic acid (TCA) were added. The solution was homogenised for 30 s at 13,000 rpm using a T25 digital Ultraturrax (IKA^®^-Werke GmbH & Co. KG, Mindelheim, Germany). Subsequently, samples were filtered via Whatman filter paper #1 (Cytiva, Ireland). A total of 3.5 mL of filtrate was transferred to a 10 mL screw cap tube and 1.5 mL of Thiobarbituric acid was added. Samples were heated at 70 °C for 30 min and subsequently cooled on ice for 15 min. Absorbance was measured at 532 nm using a Cary 300 Bio UV-Visible spectrophotometer (Agilent Technologies, Santa Clara, CA, USA). The results were expressed as mgMDA/kg. Triplicate analysis was performed per recipe, per stage.

Texture Profile Analysis (TPA)

TPA was performed at room temperature (~15 °C) using the TA.TxT2i Stable Micro Systems Texture Analyzer (Stable Microsystems Ltd., Surrey, England) in accordance with the parameters described by [43]. Casings were removed and the pepperoni were cut into cylindrical sections 1.5 × 2 × 2 cm. A 25 kg load cell was applied with a 2 mm/s crosshead speed with 5 s between compression cycles. Hardness (N), Adhesiveness, Cohesiveness, Springiness, Resilience and Chewiness were quantified. Ten replicates were examined per recipe, with the five most inconsistent measurements discarded (*n* = 5), per triplicate batch (*n* = 15).

#### 2.4.2. Microbiological Methods

Microbiological analysis of the pepperoni products was conducted to assay and quantify changes in microbial counts (log_10_ CFU/g) in a range of indicator species in response to changes in recipe hurdles. The species assayed were selected to assess a broad range of microflora, including (i) general flora (TVC), (ii) fermentation species (Lactic Acid Bacteria (LAB)), (iii) Gram-positive spoilage species (*Staphylococcus* spp. and *Listeria* spp.), (iv) Gram-negative spoilage species (*Enterobacteriaceae* and coliforms), and (v) shelf-life indicator organisms (*Pseudomonas* spp., *B. thermosphacta* and yeasts and moulds). The resulting microbiological findings of this study are not definitive but provide indicative data towards the implications that hurdle effects have on the species studied.

##### Sample Preparation

The microbiological analysis of pepperoni was conducted using aseptic sample preparation followed by homogenization to ensure an even distribution of microorganisms. Sterile forceps and a sterile scalpel were used to aseptically dissect a 10 g transection of pepperoni, which was accurately weighed into a sterile stomacher bag (Interscience, Saint Nom la Bretèche, France) to which 90 mL of maximum recovery diluent (MRD) (Sigma-Aldrich) pre-warmed to 30 °C was added. This resulted in a 1:10 dilution, facilitating microbial recovery and standardisation of results [44]. The sample solution was homogenised thoroughly in a stomacher for 60 s (Interscience, Saint Nom la Bretèche, France). After homogenization, the sample was used for serial dilutions or direct plating onto appropriate microbiological media, depending on the target microorganisms.

#### 2.4.3. Serial Dilution

The extraction and serial dilution of the sample solution were performed aseptically to ensure accuracy and prevent contamination. Using a sterile pipette tip, 1 mL of the homogenised sample solution was extracted from the stomacher bag (Interscience, Saint Nom la Bretèche, France). The sample was then transferred into a sterile test tube, ensuring that the pipette tip did not touch the rim or contents of the tube. The test tube was vortexed thoroughly to achieve uniform dispersion of microorganisms within the suspension.

Following vortexing, the pipette tip was changed to prevent cross-contamination before proceeding with serial dilutions. For each dilution step, 1 mL of the sample was transferred into a sterile test tube containing 9 mL of pre-sterilised MRD, achieving a 1:10 dilution. This process was repeated sequentially, ensuring that a fresh pipette tip was used for each transfer, until the final dilution of 10^−9^ was reached. The resulting dilution series was then ready for microbial enumeration through plating on appropriate microbiological media [45].

##### Plating Techniques

Spread Plate

The standard spread plate technique was applied consistently across all spread-plated analyses in this study [46].

Using a sterile pipette tip, 0.1 mL of the appropriate sample dilution was aseptically pipetted onto the surface of a pre-poured and solidified selective agar plate (Sarstedt—Nümbrecht, Germany). A disposable spreader (Sarstedt—Nümbrecht, Germany) was then used to evenly distribute the inoculum across the agar surface by gently rotating the plate while spreading the sample to ensure uniform microbial dispersion. Plates were left undisturbed for 5–10 min at room temperature to allow the inoculum to absorb into the agar. Plates were then inverted and incubated at the respective temperature and duration for each selective species, as outlined in Table 2. Following incubation, colonies were enumerated and recorded as colony-forming units per gram (CFU/g) of the original sample.

##### Compact Dry Plates (Total Viable Count (TVC), Enterobacteriaceae (ETB))

The plating of the serially diluted sample onto CompactDry TC™ and CompactDry ETB™ plates was performed aseptically to ensure accurate enumeration of Total Viable Count (TVC) and Enterobacteriaceae, respectively. Using a sterile pipette tip, 1 mL of the selected dilution was carefully extracted from the dilution series and dispensed onto the centre of the CompactDry™ plate (Nissui Pharmaceutical Co., Ltd., Ueno, Japan). The sample was allowed to naturally diffuse across the plate’s surface, ensuring uniform distribution of microorganisms without the need for manual spreading. A new sterile pipette tip was then used to extract 1 mL of the same dilution, which was subsequently plated onto the CompactDry ETB plate for the enumeration of Enterobacteriaceae. Like the TC plate, the sample slowly diffused over the medium to ensure consistent microbial distribution. Following inoculation, the plates were incubated under conditions specific to the target microorganisms. The CompactDry TC plates were incubated at 35–37 °C for 24–48 h to allow for growth and quantification, while the CompactDry ETB plates were incubated at 37 °C for 24 h, enabling the selective growth of Enterobacteriaceae. After incubation, colony-forming units (CFUs) were counted based on manufacturer guidelines and recorded for further analysis [47].

##### Pour Plate Technique

The quantification of LAB and *Pediococcus* spp. was carried out by quantification via pour plate on MRS agar (Sigma-Aldrich) in alignment with [48]. *Pediococcuss* spp. were selected for via pH adjustment to 5.5 with 0.1 M HCl while stirring with a magnetic stirring bar [49]. Molten MRS Agar (250 mL) was transferred to a water-bath (Julabo SW23-MSE supplies, Tucson, AZ, USA) to be held at 45–50 °C. A 1 mL sample solution was pipetted into a sterile Petri dish. Approximately 15–20 mL of molten MRS Agar (45–50 °C) was poured into the dish. The agar was swirled in a figure-of-eight pattern for 30 s to ensure homogenous colony distribution, then allowed to cool. Once set, plates were overlaid with a second layer of agar. The plates were incubated at 37 °C ± 1 °C for 72 h. Colonies were counted and expressed as colony-forming units per gram (CFU/g) of the original sample.

### 2.5. Statistical Analysis

All statistical analyses were performed using SPSS (Version 28.0, IBM Corp., Armonk, NY, USA) [50]. Statistical significance was defined at *p* < 0.05 unless otherwise stated. Because biological and physicochemical conditions differ substantially across processing stages, statistical analyses were conducted separately for the pre-fermentation, post-fermentation, post-heat treatment, and post-drying stages. At each stage, only formulation factors that were biologically relevant at that point in processing were included in the model. Accordingly, salt level and nitrite level were included at all stages; heat treatment was included only at the post-heat and post-drying stages; and water activity was included only at the post-drying stage.

For each stage, dependent variables were grouped into biologically coherent parameter sets: proximate composition (moisture, fat, protein, ash); physicochemical environment (pH, lactic acid content, nitrate and TBARS); fermentation-associated microbiology (TVC and LAB); and quality/spoilage-associated microbiology * (*Staphylococcus* spp., Coliforms, *Enterobacteriaceae*, *Pseudomonas* spp., *B. thermosphacta* and Yeasts and moulds). Multivariate analysis of variance (MANOVA) was performed to assess the effects of formulation factors and any two-way interactions on grouped dependent variables. Pillai’s Trace was used as the primary multivariate test statistic due to its robustness to deviations from multivariate normality and homogeneity of covariance.

Upon detection of significant multivariate effects, follow-up univariate general linear models were conducted for individual dependent variables. All two-way interactions were evaluated; however, salt × nitrite was the only interaction consistently retained following multivariate testing. To support interpretation of these findings within the fractional factorial design, exploratory one-way analyses of variance (ANOVA) were conducted for individual recipe factors at each stage. In addition, factor-level descriptive statistics (mean ± standard error) were examined to identify trends across processing stages. These analyses were used as interpretive support and do not represent independent estimates of factor effects. To control for multiple comparisons within each parameter group and stage, false discovery rate (FDR) correction was applied using the Benjamini–Hochberg procedure. Only effects remaining significant after FDR adjustment were interpreted in the main text (apart from spoilage flora *). Effect sizes were reported as partial eta squared (η^2^) for multivariate analyses and epsilon squared (ε^2^) for univariate models where appropriate. Model assumptions were assessed prior to analysis; microbial count data were log-transformed to improve normality and variance homogeneity. For all parametric groups except for spoilage microbiology, statistical analysis and descriptive data were produced from replicate-level data (RLD) resulting from three independent batches per formulation, with replicate analytical measurements within each batch. Due to the high degree of between-batch heterogeneity observed for spoilage flora measurements, spoilage microbiology datasets were analysed using batch-level mean (BMD) values to minimise pseudoreplication and avoid artificial inflation of significance and effect size estimates. Furthermore, extreme divergence between spoilage species following heat treatment, together with widespread non-detect observations at the post-drying stage, resulted in limited residual variance and reduced interpretive value for multivariate analysis at these stages. Consequently, post-heat treatment and post-drying spoilage flora outcomes were interpreted primarily using descriptive statistics and supporting univariate analyses following FDR correction. 

## 3. Results

### 3.1. Stage-Specific Multivariate Effects

#### 3.1.1. Pre-Fermentation (Multivariate Effects)

At the pre-fermentation stage, multivariate analysis revealed that nitrite level was significantly associated with variation in proximate composition (Pillai’s Trace = 0.14, F(4,89) = 3.6, *p* = 0.009, η^2^ = 0.14), while the effect of salt level was not statistically significant (Pillai’s Trace = 0.09, F(4,89) = 2.2, *p* = 0.078, η^2^ = 0.09) (Table 3). A significant interaction between salt and nitrite was also observed for proximate parameters (Pillai’s Trace = 0.11, F(4,89) = 2.7, *p* = 0.038, η^2^ = 0.11). For physicochemical environmental parameters, nitrite level again showed a significant multivariate effect (Pillai’s Trace = 0.16, F(5,88) = 3.3, *p* = 0.009, η^2^ = 0.16), whereas salt level (*p* = 0.067) and the salt × nitrite interaction (*p* = 0.413) were not statistically significant. In contrast, both salt and nitrite exerted significant effects on the general and fermentation microbiota. Salt level was associated with multivariate variation (Pillai’s Trace = 0.16, F(2,43) = 4.0, *p* = 0.024, η^2^ = 0.16), while nitrite showed a stronger effect (Pillai’s Trace = 0.28, F(2,43) = 8.5, *p* = 0.001, η^2^ = 0.28). Notably, a highly significant interaction between salt and nitrite was observed (Pillai’s Trace = 0.72, F(2,43) = 54.8, *p* < 0.001, η^2^ = 0.72), indicating that the combined influence of these factors was a dominant driver of early stage microbial dynamics. For spoilage-associated organisms, salt level showed a significant multivariate effect (Pillai’s Trace = 0.86, F(6,9) = 9.0, *p* = 0.002, η^2^ = 0.86), whereas nitrite level (*p* = 0.115) and the interaction term (*p* = 0.142) were not statistically significant.

#### 3.1.2. Post-Fermentation (Multivariate Effects)

At the post-fermentation stage, both salt and nitrite levels were significantly associated with multivariate variation in proximate composition. Salt level showed a moderate effect (Pillai’s Trace = 0.13, F(4,89) = 3.4, *p* = 0.010, η^2^ = 0.13), while nitrite level exerted a stronger influence (Pillai’s Trace = 0.26, F(4,89) = 7.7, *p* < 0.001, η^2^ = 0.26) (Table 3). A significant interaction between salt and nitrite was also observed (Pillai’s Trace = 0.13, F(4,89) = 3.3, *p* = 0.010, η^2^ = 0.13), indicating that combined formulation effects contributed to compositional variation following fermentation. In contrast, no significant multivariate effects of salt (*p* = 0.320) or nitrite (*p* = 0.270) were detected for physicochemical environmental parameters. However, a significant interaction between salt and nitrite was observed (Pillai’s Trace = 0.13, F(5,88) = 2.7, *p* = 0.020, η^2^ = 0.13), suggesting that combined hurdle effects influenced environmental conditions despite the absence of independent main effects. For general and fermentation microbiota, both salt and nitrite showed significant multivariate associations. Salt level (Pillai’s Trace = 0.18, F(2,43) = 4.7, *p* = 0.010, η^2^ = 0.18) and nitrite level (Pillai’s Trace = 0.24, F(2,43) = 6.8, *p* < 0.001, η^2^ = 0.24) were associated with variation in microbial populations. In addition, a strong interaction between salt and nitrite was observed (Pillai’s Trace = 0.61, F(2,43) = 33.4, *p* < 0.001, η^2^ = 0.61), indicating that their combined effects were a major driver of fermentation microbiology. For spoilage-associated organisms, nitrite level showed a dominant multivariate effect (Pillai’s Trace = 0.98, F(6,5) = 45.7, *p* < 0.001, η^2^ = 0.98), alongside a similarly strong interaction between salt and nitrite (Pillai’s Trace = 0.97, F(6,5) = 26.4, *p* < 0.001, η^2^ = 0.97). The effect of salt alone was not statistically significant (*p* = 0.080), although a large effect size was observed (η^2^ = 0.82).

#### 3.1.3. Post-Heat Treatment (Multivariate Effects)

At the post-heat treatment stage, multivariate analysis indicated that neither salt (Pillai’s Trace = 0.00, F(3,86) = 0.1, *p* = 0.980, η^2^ = 0.00) nor nitrite level (Pillai’s Trace = 0.04, F(3,86) = 1.1, *p* = 0.340, η^2^ = 0.04) was significantly associated with variation in proximate composition (Table 3). Heat treatment showed a near-significant effect (Pillai’s Trace = 0.13, F(6,174) = 2.1, *p* = 0.060, η^2^ = 0.07), suggesting a moderate contribution to compositional variation at this stage. For physicochemical environmental parameters, heat treatment exerted a significant multivariate effect (Pillai’s Trace = 0.18, F(6,174) = 2.8, *p* = 0.010, η^2^ = 0.09), while neither salt (*p* = 0.170) nor nitrite (*p* = 0.800) showed significant independent effects. In contrast, very strong multivariate effects were observed for all three factors on general and fermentation microbiota. Salt level (Pillai’s Trace = 0.96, F(2,39) = 426.2, *p* < 0.001, η^2^ = 0.96), nitrite level (Pillai’s Trace = 0.90, F(2,39) = 175.2, *p* < 0.001, η^2^ = 0.90), and heat treatment (Pillai’s Trace = 0.81, F(4,80) = 13.6, *p* < 0.001, η^2^ = 0.41) were all strongly associated with microbial variation. Notably, no significant interaction effects between salt and nitrite were observed at this stage, indicating that the effects of individual hurdles were expressed independently following heat treatment.

#### 3.1.4. Post-Drying (Final Product) Stage (Multivariate Effects)

At the post-drying stage, all three factors, salt, nitrite, and heat treatment, were significantly associated with multivariate variation in proximate composition. Salt level (Pillai’s Trace = 0.24, F(4,85) = 6.8, *p* < 0.001, η^2^ = 0.24), nitrite level (Pillai’s Trace = 0.23, F(4,85) = 6.5, *p* < 0.001, η^2^ = 0.23), and heat treatment (Pillai’s Trace = 0.25, F(4,85) = 7.0, *p* < 0.001, η^2^ = 0.25) all exerted moderate and comparable multivariate effects (Table 3). For physicochemical environmental parameters, both salt (Pillai’s Trace = 0.22, F(5,84) = 4.7, *p* < 0.001, η^2^ = 0.22) and nitrite (Pillai’s Trace = 0.35, F(5,84) = 9.2, *p* < 0.001, η^2^ = 0.35) were significantly associated with variation, while heat treatment did not show a statistically significant effect (*p* = 0.100). In terms of colour attributes, nitrite level (Pillai’s Trace = 0.13, F(3,86) = 4.3, *p* = 0.010, η^2^ = 0.13) and heat treatment (Pillai’s Trace = 0.23, F(3,86) = 8.7, *p* < 0.001, η^2^ = 0.23) showed significant multivariate effects, whereas salt level was not significant (*p* = 0.300). For textural properties, nitrite level (Pillai’s Trace = 0.25, F(7,82) = 4.0, *p* < 0.001, η^2^ = 0.25) and heat treatment (Pillai’s Trace = 0.16, F(7,82) = 2.3, *p* = 0.040, η^2^ = 0.16) were significantly associated with variation, while the effect of salt approached significance (*p* = 0.060). Very strong multivariate effects were observed for fermentation-associated microbiota across all three factors. Salt (Pillai’s Trace = 0.91, F(2,39) = 186.3, *p* < 0.001, η^2^ = 0.91), nitrite (Pillai’s Trace = 0.87, F(2,39) = 130.9, *p* < 0.001, η^2^ = 0.87), and heat treatment (Pillai’s Trace = 0.58, F(2,39) = 26.9, *p* < 0.001, η^2^ = 0.58) were all strongly associated with microbial variation at this stage.

### 3.2. Univariate Effects Following Multivariate Analysis

#### 3.2.1. Pre-Fermentation (Multivariate Effects)

Univariate analysis following multivariate modelling identified several significant effects of salt and nitrite on physicochemical and microbial parameters at the pre-fermentation stage (Table 4 and Table 5). Salt level was associated with decreased lactic acid concentration (F(1,94) = 7.4, *p* = 0.010, ε^2^ = 0.07) and reductions in total viable counts (TVC; F(1,46) = 8.3, *p* = 0.010, ε^2^ = 0.16), lactic acid bacteria (LAB; F(1,46) = 4.5, *p* = 0.040, ε^2^ = 0.09), and coliforms (F(1,30) = 11.2, *p* < 0.001, ε^2^ = 0.44). Nitrite level was associated with decreased moisture content (F(1,94) = 7.6, *p* = 0.010, ε^2^ = 0.08), increased nitrate concentration (F(1,94) = 14.8, *p* < 0.001, ε^2^ = 0.14), and reductions in TVC (F(1,46) = 5.6, *p* = 0.020, ε^2^ = 0.11). Significant interaction effects between salt and nitrite were observed for several microbial parameters, including TVC (F(1,46) = 5.4, *p* = 0.020, ε^2^ = 0.11), LAB (F(1,46) = 76.5, *p* < 0.001, ε^2^ = 0.63), and coliforms (F(1,30) = 8.9, *p* = 0.010, ε^2^ = 0.39), indicating that the combined influence of these factors strongly affected early stage microbial populations. No *L. monocytogenes* or *Listeria* spp. were detected at the pre-fermentation stage in any product manufactured in this study.

#### 3.2.2. Post-Fermentation (Univariate Effects)

Compared to the pre-fermentation stage, the influence of nitrite and its interaction with salt became more pronounced following fermentation, particularly for microbial populations (Table 6). Salt level was associated with increased ash content (F(1,94) = 10.4, *p* < 0.001, ε^2^ = 0.10) and reductions in lactic acid bacteria (LAB; F(1,46) = 9.0, *p* < 0.001, ε^2^ = 0.17) and coliforms (F(1,28) = 14.0, *p* < 0.001, ε^2^ = 0.58). Nitrite level was associated with decreased moisture content (F(1,94) = 31.6, *p* < 0.001, ε^2^ = 0.26) and substantial reductions in *Staphylococcus* spp. (F(1,25) = 44.8, *p* < 0.001, ε^2^ = 0.82). Significant interaction effects between salt and nitrite were observed for multiple parameters, including lactic acid concentration (F(1,94) = 8.9, *p* < 0.001, ε^2^ = 0.09), total viable counts (TVC) (F(1,46) = 8.5, *p* = 0.010, ε^2^ = 0.16), LAB (F(1,46) = 46.8, *p* < 0.001, ε^2^ = 0.52), and *Staphylococcus* spp. (F(1,25) = 32.2, *p* < 0.001, ε^2^ = 0.76), indicating that combined formulation effects strongly influenced microbial and fermentation-related outcomes.

To support the interpretation of these findings, exploratory one-way comparisons were examined. These analyses showed expected trends, including strong associations between salt level and product salt concentration (F(1,94) = 311.9, *p* < 0.001, ε^2^ = 0.77) and between nitrite level and residual nitrite concentration (F(1,94) = 1858.5, *p* < 0.001, ε^2^ = 0.95). Additional associations were observed between nitrite level and TBARS (F(1,94) = 6.3, *p* = 0.010, ε^2^ = 0.06), *Staphylococcus* spp. (F(1,25) = 12.9, *p* < 0.001, ε^2^ = 0.34), and *Enterobacteriaceae* (F(1,30) = 14.1, *p* < 0.001, ε^2^ = 0.32). However, these comparisons are presented as descriptive support and do not represent independent estimates of factor effects. All reported effects remained significant following Benjamini–Hochberg FDR correction. No *L. monocytogenes* or *Listeria* spp. were detected at the post-fermentation stage in any product manufactured in this study.

#### 3.2.3. Post-Heat Treatment (Univariate Effects)

At the post-heat treatment stage, extensive univariate effects of salt, nitrite, and heat treatment were revealed on microbial and physicochemical parameters (Table 7). Salt level was associated with substantial reductions in general and fermentation microbial populations, total viable counts (TVC; F(1,46) = 653.7, *p* < 0.001, ε^2^ = 0.94), and lactic acid bacteria (LAB; F(1,46) = 168.3, *p* < 0.001, ε^2^ = 0.81). Nitrite level similarly exhibited strong associations with its reduction, TVC (F(1,46) = 302.8, *p* < 0.001, ε^2^ = 0.88), and LAB (F(1,46) = 105.4, *p* < 0.001, ε^2^ = 0.72). Heat treatment exerted significant effects on both physicochemical and microbial parameters. Lactic acid concentration (F(1,94) = 8.2, *p* = 0.001, ε^2^ = 0.16) and TVC (F(1,46) = 11.1, *p* < 0.001, ε^2^ = 0.36) were significantly reduced.

Examination of additional one-way comparisons showed consistent trends, including strong associations between salt level and lactic acid concentration (F(1,94) = 470.1, *p* < 0.001, ε^2^ = 0.83), TVC (F(1,46) = 24.2, *p* < 0.001, ε^2^ = 0.34), and LAB (F(1,46) = 14.5, *p* < 0.001, ε^2^ = 0.24). Salt was found to have no significant effect on spoilage flora at this stage. Nitrite was found to significantly reduce *Staphylococcus* spp. (F(1,20) = 21.8, *p* < 0.001, ε^2^ = 0.50), while heat treatment was found to have the most significant effect on reducing spoilage-associated species, with significant effects for *Enterobacteriaceae* (F(2,17) = 111.3, *p* < 0.001, ε^2^ = 0.92)*, Pseudomonas* spp. (F(2,17) = 10.9, *p* = 0.001, ε^2^ = 0.52) and *B. thermosphacta* (F(2,18) = 6.1, *p* = 0.010, ε^2^ = 0.34). Yeasts and moulds were fully eliminated at this stage; therefore, no univariate findings were produced. These comparisons are presented as descriptive support and do not represent independent estimates of factor effects. All reported effects remained significant following Benjamini–Hochberg FDR correction. As with both prior stages, no *L. monocytogenes* or *Listeria* spp. were detected in any recipe.

#### 3.2.4. Post-Drying (Univariate Effects)

At the post-drying stage, univariate effects of the multivariate model identified significant effects of salt, nitrite, and heat treatment on compositional, microbial, and quality-related parameters (Table 8). Salt level was associated with increased fat (F(1,94) = 8.1, *p* = 0.005, ε^2^ = 0.08) and ash content (F(1,94) = 17.7, *p* < 0.001, ε^2^ = 0.17), as well as reductions in lactic acid concentration (F(1,94) = 20.9, *p* < 0.001, ε^2^ = 0.19). Strong associations were also observed for microbial parameters, including TVC (F(2,45) = 115.7, *p* < 0.001, ε^2^ = 0.74) and lactic acid bacteria LAB (F(2,45) = 356.5, *p* < 0.001, ε^2^ = 0.90). Nitrite level was associated with multiple compositional and quality-related changes, including increased fat content (F(1,94) = 22.1, *p* < 0.001, ε^2^ = 0.20), reduced lactic acid concentration (F(1,94) = 10.3, *p* = 0.002, ε^2^ = 0.11), and increased nitrate concentration (F(1,94) = 23.8, *p* < 0.001, ε^2^ = 0.21). Significant effects were also observed for TBARS (F(2,93) = 9.4, *p* = 0.003, ε^2^ = 0.10), colour (a*; F(2,93) = 13.1, *p* < 0.001, ε^2^ = 0.13), and textural properties including cohesiveness (F(1,94) = 17.2, *p* < 0.001, ε^2^ = 0.16) and resilience (F(1,94) = 16.2, *p* < 0.001, ε^2^ = 0.16). Nitrite also showed strong associations with reductions in general and fermentation microbial populations, TVC (F(1,46) = 65.7, *p* < 0.001, ε^2^ = 0.62), and LAB (F(1,46) = 240.8, *p* < 0.001, ε^2^ = 0.86). Heat treatment exerted significant effects on moisture (F(1,94) = 10.3, *p* = 0.002, ε^2^ = 0.10), fat (F(1,94) = 19.0, *p* < 0.001, ε^2^ = 0.18), colour (b*; F(2,93) = 14.5, *p* < 0.001, ε^2^ = 0.14), and microbial populations, including TVC (F(2,45) = 49.8, *p* < 0.001, ε^2^ = 0.55) and LAB (F(2,45) = 14.0, *p* = 0.001, ε^2^ = 0.26).

Additional one-way comparisons revealed broader associations across compositional and textural parameters, including effects of salt on moisture, protein, pH, and texture attributes, and effects of nitrite on colour parameters (L*, a*, b*) and residual curing agent concentrations. Heat treatment showed consistent associations with physicochemical properties, including moisture, fat, protein and lipid oxidation (TBARS), as well as multiple texture attributes. Salt and heat treatment were found to be significantly influential in the suppression of *Pseudomonas* spp. in the dry stage; (Salt: (F(1,22) = 11, *p* = 0.003, ε^2^ = 0.30), Heat treatment: (F(2,21) = 13.1, *p* < 0.001, ε^2^ = 0.51)), while nitrite was found to significantly suppress *B. thermosphacta* (F(1,21) = 8.7, *p* = 0.008, ε^2^ = 0.26) in the dry stage.

In addition, water activity (aW), which was not retained as an independent factor in the multivariate model, showed associations with compositional and textural properties, including moisture, fat, protein, adhesiveness, cohesiveness, gumminess, chewiness, and resilience. Water activity had no significant influence on microbial species. These exploratory comparisons are presented as descriptive support and do not represent independent estimates of factor effects. All reported effects remained significant following Benjamini–Hochberg FDR correction. No *L. monocytogenes* or *Listeria* spp. were detected in any recipe at the dry stage.

### 3.3. Factor-Level Descriptive Trends Across Processing Hurdles

#### 3.3.1. Salt

To support interpretation of the multivariate and univariate analyses, factor-level descriptive trends for salt concentration were examined across all processing stages (Table 9). The comparisons presented are all parameters that were significant following FDR correction, in both multivariate and univariate analyses, except for spoilage flora, for which all findings are presented. At the pre-fermentation stage, higher salt levels (2.5%) were associated with slightly lower moisture content and increased protein and ash concentrations relative to the lower salt formulation (1.4%). Microbial counts were consistently reduced at higher salt levels, including total viable counts (TVC), lactic acid bacteria (LAB), and coliforms, indicating an early inhibitory effect of salt on microbial populations. Following fermentation, these trends persisted, with higher salt levels associated with reduced microbial counts and increased ash and salt concentrations. Differences in lactic acid concentration between salt levels were minimal at this stage, suggesting comparable fermentation activity across formulations. After heat treatment, the inhibitory effect of salt on microbial populations became more pronounced. Higher salt levels were associated with substantially lower counts of TVC, LAB, *Staphylococcus* spp., coliforms, *Enterobacteriaceae*, *Pseudomonas* spp. and *B. thermosphacta*, indicating a strong combined effect of salt and thermal processing on microbial reduction.

At the post-drying stage, salt level was associated with clear compositional and structural differences. Higher salt formulations showed reduced moisture content and increased fat, protein, and ash concentrations, consistent with concentration effects arising from differential water loss. Textural parameters, including hardness and gumminess, were also higher at increased salt levels, indicating a firmer product structure. Microbial differences between salt levels persisted following drying, with lower counts of TVC and LAB. All spoilage-associated organisms were reduced in higher salt formulations with *Pseudomonas* spp. retaining significance following FDR correction. These findings suggest that salt contributes to both microbial control and structural development throughout processing, with effects becoming increasingly pronounced following heat treatment and drying.

#### 3.3.2. Nitrite

To support interpretation of the multivariate and univariate analyses, factor-level descriptive trends for nitrite concentration were examined across all processing stages (Table 10). These comparisons are presented as descriptive support and do not represent independent estimates of factor effects due to the fractional factorial design.

At the pre-fermentation stage, higher nitrite levels (150 ppm) were associated with lower moisture content and increased residual nitrate and nitrite concentrations compared to the lower nitrite formulation (50 ppm). Microbial counts, including total viable counts (TVC) and coliforms, were marginally lower at increased nitrite levels at this stage, suggesting limited early antimicrobial impact prior to fermentation. Following fermentation, differences between nitrite levels became more pronounced. Higher nitrite concentrations were associated with increased residual nitrate and nitrite levels and elevated lipid oxidation (TBARS), while reductions in microbial populations, particularly *Staphylococcus* spp., coliforms and *Enterobacteriaceae*, began to emerge. After heat treatment, the antimicrobial effects of nitrite were clear. Higher nitrite formulations showed substantial reductions in microbial populations, including *Staphylococcus* spp. and coliforms, and the elimination of yeasts and moulds, indicating a strong combined effect of nitrite and thermal processing on microbial inactivation.

At the post-drying stage, nitrite level was associated with pronounced differences in compositional, microbial, and quality attributes. Higher nitrite formulations exhibited increased fat and protein concentrations and reduced lactic acid levels, consistent with concentration and curing effects. Big differences were also observed in residual nitrate and nitrite concentrations, reflecting curing agent retention throughout processing. Nitrite level was further associated with colour development, with higher values of L*, a*, and b* observed at increased nitrite levels, indicating enhanced cured colour formation. In addition, textural attributes, including cohesiveness and resilience, were elevated in higher nitrite formulations. Microbial differences persisted following drying, with lower counts of TVC LAB, *Enterobacteriaceae* and *Pseudomonas* spp. Both coliforms and *B. thermosphacta* had no detectible counts in elevated nitrite formulations, and *Staphylococcus* spp. and yeasts and moulds were both eliminated in the dry stage across all formulations. These findings indicate that nitrite contributes to microbial control, curing chemistry, and product quality development, with effects becoming increasingly pronounced following heat treatment and drying.

#### 3.3.3. Heat Treatment

To support interpretation of the multivariate and univariate analyses, factor-level descriptive trends for heat treatment were examined across the post-heat and post-drying stages (Table 11). These comparisons are presented as descriptive support and do not represent independent estimates of factor effects due to the fractional factorial design.

At the post-heat treatment stage, clear differences were observed between the three heat treatments (53 °C/60 min, 61 °C/40 min, and 64 °C/20 min). While physicochemical parameters such as pH and water activity (aW) remained largely similar across treatments, differences in lactic acid concentration were evident, with the highest value observed under the 64 °C/20 min treatment. Microbial responses varied substantially between heat treatments. The highest temperature treatment (64 °C/20 min) was associated with the greatest reductions in microbial populations, including complete or near-complete reductions in coliforms, *Enterobacteriaceae*, *Pseudomonas* spp., and yeasts and moulds. In contrast, intermediate treatment conditions (61 °C/40 min) were associated with higher residual counts of several microbial groups, including TVC, LAB, and spoilage-associated organisms, indicating reduced microbial inactivation relative to the higher temperature treatment.

At the post-drying stage, the effects of heat treatment on product composition and quality became more pronounced. Differences in moisture content were evident, with the intermediate heat treatment (61 °C/40 min) associated with higher residual moisture compared to the lower and higher temperature treatments. Correspondingly, compositional parameters such as fat, protein, and ash varied across treatments, suggesting differences in moisture loss and concentration effects during drying. Heat treatment also influenced oxidative stability, with TBARS values highest in the intermediate treatment (61 °C/40 min) and lowest in the highest temperature treatment (64 °C/20 min), indicating differential lipid oxidation responses across time–temperature combinations. Clear differences in colour development were observed, with the highest redness (a*) associated with the 64 °C/20 min treatment, while the 61 °C/40 min treatment exhibited lower L*, a*, and b* values, indicating reduced colour intensity. Texture was also affected, with increasing treatment severity associated with more negative adhesiveness values, suggesting a more adhesive product structure. Microbial differences between treatments persisted following drying. The highest temperature treatment (64 °C/20 min) consistently resulted in the lowest microbial counts across multiple taxa, including TVC, LAB, *Enterobacteriaceae*, and *Pseudomonas* spp., while the intermediate treatment retained higher microbial loads. These findings indicate that heat treatment plays a critical role in microbial reduction, with higher temperature, shorter time combinations associated with greater microbial inactivation.

#### 3.3.4. Water Activity (aW)

Factor-level descriptive trends of the multivariate and univariate analyses for water activity (aW) (0.91 vs. 0.94) revealed clear compositional differences in the final product (Table 12). Lower aW (0.91) was associated with reduced moisture content and correspondingly higher fat, protein, and salt concentrations, consistent with increased drying and concentration effects. In contrast, higher aW (0.94) retained greater moisture and lower concentrations of these components. Water activity was also associated with differences in oxidative stability, with lower aW formulations exhibiting reduced TBARS values compared to higher aW formulations, indicating lower levels of lipid oxidation under drier conditions. Textural properties were notably influenced by aW. Lower aW (0.91) was associated with increased cohesiveness, gumminess, chewiness, and resilience, indicating a firmer and more structured product. In contrast, a higher aW (0.94) resulted in more negative adhesiveness values, suggesting a softer and more adhesive texture. These findings indicate that while aW was not retained as an independent factor in the multivariate model, it was associated with meaningful differences in product composition, oxidative stability, and texture at the final stage. This supports its role as a secondary or interacting factor within the overall hurdle system, primarily reflecting the effects of moisture loss during drying rather than acting as an independent driver of variation.

## 4. Discussion

### 4.1. Stage-Dependent Roles of Salt, Nitrite, Heat Treatment, and Water Activity as Processing Hurdles

The present study demonstrates that the effects of processing hurdles in fermented meat manufacture are strongly stage-dependent, with salt, nitrite, heat treatment, and water activity contributing differently across successive stages of production. Rather than acting as static or additive factors, these hurdles exhibited dynamic and evolving roles, reflecting both physicochemical changes and microbial responses throughout processing.

At the pre-fermentation stage, the effects of individual hurdles were present but limited in magnitude. Salt was associated with modest reductions in microbial populations, including total viable counts (TVC), lactic acid bacteria (LAB), and coliforms, indicating an early inhibitory effect [5]. In contrast, nitrite did not exhibit a clear antimicrobial effect at this stage, and in some cases, microbial counts were not reduced at higher nitrite levels [51]. However, interaction effects between salt and nitrite were already evident, suggesting that combined hurdle effects begin to emerge prior to fermentation [7]. These findings indicate that early microbial structuring provides the baseline upon which subsequent fermentation dynamics develop.

Following fermentation, the combined action of salt and nitrite became increasingly important, with the salt × nitrite interaction emerging as the dominant regulator of fermentation intensity, with the divergence in their independent hurdle effects becoming more apparent [52]. Salt continued to be associated with reductions in microbial populations, while nitrite began to exhibit a more pronounced antimicrobial role, particularly against *Staphylococcus* spp. and *Enterobacteriaceae*. The persistence of interaction effects between salt and nitrite at this stage suggests that combined hurdle action contributes to microbial control during fermentation [53]. At the same time, acidification progressed similarly across formulations, indicating that although LAB were limited by salt and nitrite, their halotolerance allowed their proliferation to persist, with fermentation itself a dominant driver of microbial and physicochemical change [54,55]. In contrast, spoilage and quality-associated flora lacked halotolerance while also displaying sensitivity to pH, hence their reduction [56]. This finding is consistent with hurdle theory, whereby combined sub-lethal stresses exert greater regulatory influence than individual factors acting alone [57].

The post-heat treatment stage represented the most significant point of microbial reduction within the process. Heat treatment was associated with substantial decreases in microbial populations across all measured groups, including spoilage-associated organisms and indicator bacteria. Importantly, the effects of heat treatment were not uniform across time–temperature combinations. The highest temperature treatment (HT3: 64 °C/20 min) was consistently associated with the lowest microbial counts, while the intermediate treatment (HT2: 61 °C/40 min) retained the highest residual populations, indicating that temperature intensity was a significant driver in microbiological lethality. However, the lowest temperature heat treatment (HT1: 53 °C/60 min) was found to be the second most effective heat-treatment in microbiological lethality, indicating that extended duration effectively mitigated the effect of high temperature, with less physicochemical impact. This non-linear response highlights the importance of achieving the optimal time-temperature ratio in treatment, determining microbial inactivation and physicochemical characteristics [58,59]. While salt and nitrite continued to be associated with reductions in microbial populations at this stage, their effects were secondary to those of heat treatment, with thermal processing representing the dominant hurdle for microbial control.

At the post-drying stage, the role of hurdles shifted from microbial inactivation towards product stabilisation and quality development. Salt was associated with reduced moisture content, which contributed to proportional increases in fat, protein, and ash concentrations through drying-induced concentration effects, which were compositional increases resulting from relative changes associated with water loss rather than absolute increases in constituent mass [60]. Nitrite contributed to curing-related changes, including increased colour intensity, reduced lipid oxidation, improved cohesiveness, alongside continued associations with reduced microbial counts [61]. Heat treatment effects persisted in both microbial and physicochemical outcomes, influencing oxidation, colour, and texture. Heat treatment imparted residual structural and microbial load effects rather than ongoing antimicrobial lethality, with the persistence of its effects largely dependent on formulation salt/ nitrite content. Water activity (aW), although not retained as an independent factor in the multivariate model, was associated with clear differences in compositional and textural properties at the final stage. Lower aW levels corresponded to reduced moisture and increased concentrations of other components, as well as firmer texture characteristics. These effects are consistent with the role of aW as a secondary and product stabilising factor reflecting drying intensity, with effects which may become more significant throughout the product’s shelf-life, rather than an independently acting hurdle at the end of processing [29].

Overall, these findings demonstrate that effective hurdle design in fermented meat systems depends not only on the presence of multiple barriers, but on their coordinated deployment of effects across processing stages. Hurdle effects were not static but evolved throughout processing, with different factors dominating at different stages. Early stage microbial dynamics were influenced primarily by the independent effects of salt and nitrite, which, in combination, produced a compound regulatory effect. Heat treatment acted as the principal microbial inactivation step, and drying consolidated product stability and quality. This stage-dependent behaviour underscores the importance of considering temporal dynamics when designing and optimising hurdle-based preservation strategies in fermented meat systems. These findings support a dynamic interpretation of hurdle technology, in which the relative contribution of individual hurdles shifts across processing stages rather than acting as fixed or additive barriers, providing a mechanistic basis for optimising salt and nitrite reduction strategies while maintaining fermentation performance and product safety.

### 4.2. Implications for Salt and Nitrite Reduction Strategies in Fermented Meat Manufacture

The stage-dependent behaviour of processing hurdles observed in this study has important implications for strategies aimed at reducing salt and nitrite levels in fermented meat products. The findings indicate that both salt and nitrite perform multiple roles that vary across processing stages and therefore cannot be reduced uniformly without consideration of their respective roles in regulating fermentation performance, microbial safety and product quality [62,63].

Salt was associated with microbial suppression at early and intermediate stages, as well as with compositional and structural changes during drying. Reductions in salt level were linked to higher microbial counts during processing and altered final product characteristics, including increased moisture content and reduced firmness. Salt likely contributed to these effects through its role in myofibrillar protein extraction, which facilitates protein solubilisation, matrix binding, and moisture migration during drying. Consequently, higher salt formulations exhibited enhanced moisture loss and developed a denser, more cohesive final product texture. These observations reflect the dual role of salt as both an antimicrobial agent and a driver of water loss and texture development [64,65]. Consequently, salt reduction strategies must account not only for microbial safety but also for its influence on drying behaviour and product structure [5,66]. Nitrite exhibited a more stage-dependent pattern of activity. Limited antimicrobial effects were observed at the pre-fermentation stage, whereas more pronounced reductions in microbial populations were evident following fermentation and which increased after heat treatment. In the dry stage, the role of nitrite expanded, becoming a key regulator of physicochemical and microbiological quality, particularly in association with curing-related attributes.

The results further highlight that the effects of salt and nitrite were interactive with processing factors. Heat treatment, particularly, emerged as a dominant determinant of microbial reduction, indicating that optimisation of thermal processing may provide a pathway to partially offset reductions in salt and nitrite [67,68]. However, the concentration of salt and nitrite was a critical factor in ensuring the persistence of heat treatment effects into the dry stage, with their presence inhibiting the reemergence of spoilage and quality-related species. Water activity also played an important role in shaping final product characteristics, although it was not independently retained in the multivariate model. Differences in aW were associated with compositional concentration and textural properties, reflecting the impact of drying intensity. This suggests that control of drying conditions may provide an additional lever for managing the effects of salt reduction, particularly in relation to texture and stability.

Taken together, these findings indicate that successful reduction in salt and nitrite in fermented meat systems requires an integrated, stage-specific approach. Rather than focusing on single-factor reductions, reformulation strategies should consider the respective effects and interactions that salt and nitrite have with other hurdles and the product matrix throughout the manufacturing process. Reductions in nitrite may be achievable with minimal impact on fermentation, whereas reductions in salt are more likely to disrupt early microbial ecology [67]. Conversely, reductions in nitrite are more likely to be impactful later in manufacture, posing greater risks to both safety and quality. Future reformulation strategies may require the incorporation of additional or alternative stabilising hurdles, such as functional starter cultures and targeted antimicrobial systems, in conjunction with modified processing conditions such as optimised heat treatment and drying regimes to maintain microbial safety and product quality [3,4].

### 4.3. Implications for Fermented Meat Safety and Hurdle Technology

The findings of this study reinforce the central principles of hurdle technology while providing new, stage-resolved insights into how individual and combined hurdles contribute to microbial safety in fermented meat systems. Rather than acting as static or redundant barriers, salt, nitrite, heat treatment, and water activity functioned as dynamically interacting hurdles whose relative importance shifted across processing stages. This temporal structuring of hurdle dominance has important implications for both risk management and product reformulation. In contrast, heat treatment emerged as the dominant factor governing microbial reduction at the post-processing stage. This sequential pattern of hurdle dominance suggests that microbial control in fermented meat manufacture is achieved through a progression of effects rather than through static additive interactions.

The study also highlights that interactions between hurdles are stage-dependent. Significant interactions between salt and nitrite were observed at early stages, indicating that combined effects contribute to microbial control prior to and during fermentation [69,70]. However, these interaction effects diminished following heat treatment, where thermal processing became the primary determinant of microbial inactivation and the respective independent effects of salt and nitrite became more defined. This finding suggests that synergy between hurdles is not uniform but instead depends on the stage of processing and the prevailing physicochemical conditions [58,71].

An important implication of these findings is that seemingly equivalent time–temperature processing conditions of heat treatment did not produce equivalent microbiological outcomes. This non-equivalence has direct relevance for process validation and highlights the need for empirical evaluation of hurdle combinations rather than reliance on theoretical equivalence. The role of water activity further illustrates the complexity of hurdle interactions. Although aW was not retained as an independent factor in the multivariate model, it was associated with differences in compositional and textural properties at the final stage. This reflects the close coupling between aW and drying and indicates that its effects could not be fully disentangled from other processing factors due to the much greater magnitude of their effects. As such, aW may function primarily as a secondary or dependent hurdle in this context, influencing product stability indirectly through its relationship with moisture loss.

Taken together, these findings support a dynamic interpretation of hurdle technology in which the relative contribution of individual factors shifts throughout processing. Rather than acting as fixed barriers, hurdles interact within a temporally evolving system, with different factors assuming primary importance at different stages. This perspective has implications for both process design and safety validation, suggesting that effective hurdle strategies must consider not only the presence of multiple factors, but also their timing, interaction, and stage-specific functionality.

### 4.4. Study Limitations and Future Research Directions

While this study provides a detailed stage-resolved analysis of processing hurdles during fermented meat manufacture, several limitations should be acknowledged when interpreting the findings. Firstly, the experimental design represented a fractional subset of the nominal factorial structure, meaning that not all main effects and interactions could be estimated independently. Particularly, relationships involving heat treatment and water activity were not fully orthogonal across formulations. Consequently, some observed responses may reflect combined or partially confounded influences of multiple processing factors, and interpretations should therefore be made within the constraints of the experimental design.

Furthermore, salt, nitrite, and water activity were each evaluated at only two levels. As such, the study was not intended to establish precise optimisation thresholds or fully characterise nonlinear response behaviour across the complete manufacturing range. Rather, the factorial structure was designed to identify the relative importance, directional behaviour, and interaction potential of key hurdle combinations under controlled pepperoni manufacturing conditions. Within this context, the findings provide practical insight into the feasibility of reducing conventional curing hurdles while maintaining microbial stability and physicochemical integrity. Nevertheless, further work incorporating intermediate factor levels or response surface methodologies would be beneficial to define precise industrial optimisation targets and refine formulation boundaries.

Secondly, although the physicochemical assessment incorporated a broad range of compositional, fermentative, curing, oxidative, colour, and textural parameters relevant to fermented meat quality and stability, additional analytical approaches may provide further mechanistic insight into formulation-driven changes. Analyses relating to protein oxidation, volatile flavour compounds, water mobility, or microstructural development may help further characterise the structural and sensory consequences of hurdle modification. However, the analytical framework employed in the present study was designed to prioritise the primary physicochemical and microbiological mechanisms governing fermented pepperoni stability and quality during processing. Microbial outcomes in this study were assessed using indicator organisms and general microbial populations, rather than through pathogen challenge testing. While these measures provide valuable insight into microbial dynamics and relative hurdle effectiveness, they do not allow direct inference of pathogen inactivation or safety under all conditions. Consequently, conclusions relating to microbial safety should be interpreted as indicative rather than definitive. The incorporation of molecular or metagenomic approaches in future studies could provide deeper insight into microbial succession, stress adaptation, and competitive interactions, particularly under reduced-salt and nitrite conditions [72].

Thirdly, the study was conducted using a single product formulation and processing framework, which may limit the generalisability of the findings to other fermented meat systems. Variations in raw materials, formulation composition, and processing conditions could influence the relative effectiveness of individual hurdles and their interactions. In addition, sensory attributes and shelf-life stability were not evaluated as part of this study. While physicochemical and microbiological parameters provide important indicators of product quality and stability, the absence of sensory and storage data limits the ability to fully assess the practical implications of formulation and processing changes. Finally, the role of water activity could not be fully resolved as an independent factor within the statistical model, due to its close coupling with drying dynamics. As such, its effects are interpreted as secondary and associated with moisture loss rather than as a fully independent hurdle.

Future research should aim to address these limitations through fully crossed experimental designs, incorporation of pathogen challenge studies, and evaluation of sensory and shelf-life outcomes. Furthermore, future research should focus on integrating functional starter cultures, alternative antimicrobial systems, and modified processing strategies to compensate for reduced salt and nitrite levels while preserving safety and quality [73,74]. Particularly, starter cultures designed to enhance acidification, pathogen suppression, or oxidative stability may provide targeted support at stages where conventional hurdles are reduced [51,75,76]. By addressing these limitations, future work can build on the mechanistic framework established here to refine hurdle design and support the development of fermented meat products that balance safety, quality, and nutritional objectives.

## 5. Conclusions

This study demonstrates that the effectiveness of salt, nitrite, heat treatment, and water activity as processing hurdles in dry fermented meat manufacture is strongly stage-dependent. Multivariate analysis revealed that nitrite and its interaction with salt were key determinants of early stage microbial dynamics (η^2^ ≤ 0.72), while heat treatment emerged as the dominant factor governing microbial reduction in late processing. Differences in heat treatment time–temperature combinations resulted in non-equivalent microbiological outcomes, highlighting the importance of process-specific validation.

At the final stage, salt and nitrite were associated with both microbial stability and product quality attributes, including composition, texture, and colour, while water activity contributed to structural and concentration-related effects despite not being independently retained in the statistical model. These findings support a dynamic interpretation of hurdle technology, in which the relative contribution of individual factors shifts throughout processing. The results provide a framework for the development of reformulation strategies aimed at reducing salt and nitrite levels, emphasising the need for integrated, stage-specific optimisation of multiple processing parameters to maintain safety and product quality.

## Figures and Tables

**Table 1:** Recipe design. Experimental formulation design showing salt level, nitrite concentration, heat treatment regime, and target water activity applied in each recipe. Salt and nitrite were incorporated at formulation, heat treatment was applied post-fermentation, and water activity was achieved during the drying stage. Batches per formulation (*n* = 3).

Recipe	Nitrite (ppm)	Salt (%)	Heat Treatment	aW
R1	150	2.5	53.5 °C for 60 min	0.91
R2	50	1.4	61 °C for 40 min	0.91
R3	150	1.4	61 °C for 40 min	0.94
R4	50	2.5	61 °C for 40 min	0.94
R5	50	1.4	64 °C for 20 min	0.94
R6	150	2.5	64 °C for 20 min	0.91
R7	50	2.5	53.5 °C for 60 min	0.91
R8	150	1.4	53.5 °C for 60 min	0.91

**Table 2:** Protocol for the screening and quantification of microbial species. The plating techniques, media and incubation conditions of the microbial species were quantified in this study.

Technique	Species	Media	Incubation
Spread plate	*Staphylococcus* spp.	Baird-Parker Agar Base ^a^ with Egg-Tellurite Emulsion ^a^	37 °C for 48–72 h
	Coliforms (inc. *E. coli*)	ECC Chromoselective Agar ^b^	37 °C for 24 h
	*Pseudomonas* spp.	Pseudomonas Agar Base ^a^ (with selective supplement ^a^)	37 °C for 24–48 h
	*B. thermosphacta*	STAA Agar Base ^a^ (with selective supplement ^a^)	20 °C for 24–48 h
	Yeasts and Moulds	Rose-Bengal Chloramphenicol Agar Base ^a^ (with 0.4% Chloramphenicol ^a^)	25 °C for 96–120 h
	*L. monocytogenes* *	*L. monocytogenes* selective media (OXFORD) ^a^ (with selective supplement ^a^)	37 °C for 24–48 h
CompactDry	TC (Total Viable Count)	CompactDry™ (TC) plates ^c^	30 °C for 24–48 h
	ETB (*Enterobacteriaceae*)	CompactDry™ (ETB) plates ^c^	37 °C for 24–48 h
Pour plate	L.A.B	(De Man-Rogosa- Sharpe) MRS Agar ^a^	37 °C for 24–48 h
	*Pediococcus* spp.	MRS Agar ^a^ (pH adjusted w/0.1 M HCl)	37 °C for 24–48 h

^a^—OXOID Ltd. Hampshire, UK. ^b^—Merck KGaA, Darmstadt, Germany. ^c^—Nissui Pharmaceutical Co. Ltd., Ueno, Japan. * Although assessed, no *L monocytogenes* or *Listeria* spp. were detected in this study; therefore, no results are presented for this species.

**Table 3:** Multivariate analysis on the effects of hurdles on measured parameters throughout the manufacturing process. A stagewise multivariate analysis on the impact of recipe hurdles on parametric subgroups throughout manufacture (RLD).

Pre-Fermentation					
Analyte Group	Recipe Factor	Pillai’s Trace	F(df1,df2)	Sig.	Partial η^2^
Proximal Composition	Salt	0.09	2.2(4,89)	0.078	0.09
	Nitrite	0.14	3.6(4,89)	0.009	0.14
	Salt × Nitrite *	0.11	2.7(4,89)	0.038	0.11
Physicochemical Environment	Salt	0.11	2.2(5,88)	0.067	0.11
	Nitrite	0.16	3.3(5,88)	0.009	0.16
	Salt × Nitrite *	0.05	1(5,88)	0.413	0.05
General and Fermentation Flora	Salt	0.16	4(2,43)	0.024	0.16
	Nitrite	0.28	8.5(2,43)	0.001	0.28
	Salt × Nitrite *	0.72	54.8(2,43)	<0.001	0.72
Spoilage Flora	Salt	0.86	9(6,9)	0.002	0.86
	Nitrite	0.62	2.4(6,9)	0.115	0.62
	Salt × Nitrite *	0.59	2.2(6,9)	0.142	0.59
Post-Fermentation					
Analyte Group	Recipe Factor	Pillai’s Trace	F(df1,df2)	Sig.	Partial η^2^
Proximal Composition	Salt	0.13	3.4(4,89)	0.012	0.13
	Nitrite	0.26	7.7(4,89)	<0.001	0.26
	Salt × Nitrite *	0.13	3.3(4,89)	0.014	0.13
Physicochemical Environment	Salt	0.06	1.2(5,88)	0.324	0.06
	Nitrite	0.07	1.3(5,88)	0.273	0.07
	Salt × Nitrite *	0.13	2.7(5,88)	0.025	0.13
General and Fermentation Flora	Salt	0.18	4.7(2,43)	0.014	0.18
	Nitrite	0.24	6.8(2,43)	0.003	0.24
	Salt × Nitrite *	0.61	33.4(2,43)	<0.001	0.61
Spoilage Flora	Salt	0.82	3.9(6,5)	0.080	0.82
	Nitrite	0.98	45.7(6,5)	<0.001	0.98
	Salt × Nitrite *	0.97	26.4(6,5)	0.001	0.97
Post-Heat Treatment					
Analyte Group	Recipe Factor	Pillai’s Trace	F(df1,df2)	Sig.	Partial η^2^
Proximal Composition	Salt	0.00	0.1(3,86)	0.982	0.00
	Nitrite	0.04	1.1(3,86)	0.336	0.04
	Heat Treatment	0.13	2.1(6,174)	0.056	0.07
Physicochemical Environment	Salt	0.06	1.7(3,86)	0.169	0.06
	Nitrite	0.01	0.3(3,86)	0.800	0.01
	Heat Treatment	0.18	2.8(6,174)	0.013	0.09
General and Fermentation Flora	Salt	0.96	426.2(2,39)	<0.001	0.96
	Nitrite	0.90	175.2(2,39)	<0.001	0.90
	Heat Treatment	0.81	13.6(4,80)	<0.001	0.41
Spoilage Flora **	Salt	-	-	-	-
	Nitrite	-	-	-	-
	Heat Treatment	-	-	-	-
Dry					
Analyte Group	Recipe Factor	Pillais Trace	F(df1,df2)	Sig.	Partial η^2^
Proximal Composition	Salt	0.24	6.8(4,85)	<0.001	0.24
	Nitrite	0.23	6.5(4,85)	<0.001	0.23
	Heat Treatment	0.25	7(4,85)	<0.001	0.25
Physicochemical Environment	Salt	0.22	4.7(5,84)	0.001	0.22
	Nitrite	0.35	9.2(5,84)	<0.001	0.35
	Heat Treatment	0.10	1.9(5,84)	0.095	0.10
Colour	Salt	0.04	1.2(3,86)	0.300	0.04
	Nitrite	0.13	4.3(3,86)	0.007	0.13
	Heat Treatment	0.23	8.7(3,86)	<0.001	0.23
Texture Profile	Salt	0.15	2(7,82)	0.062	0.15
	Nitrite	0.25	4(7,82)	0.001	0.25
	Heat Treatment	0.16	2.3(7,82)	0.037	0.16
General and Fermentation Flora	Salt	0.91	186.3(2,39)	<0.001	0.91
	Nitrite	0.87	130.9(2,39)	<0.001	0.87
	Heat Treatment	0.58	26.9(2,39)	<0.001	0.58
Spoilage Flora **	Salt	-	-	-	-
	Nitrite	-	-	-	-
	Heat Treatment	-	-	-	-

* No multivariate effects were detected beyond post-fermentation. ** Due to widespread suppression of spoilage flora following heat treatment and drying, several microbial datasets exhibited limited residual variance and high frequencies of non-detect observations, resulting in their unsuitability for multivariate analysis. Consequently, these stages were interpreted primarily using descriptive statistics and supporting one-way ANOVA analyses, with Benjamini–Hochberg FDR correction applied where appropriate.

**Table 4:** A Summary of parameters significantly affected by hurdle effects throughout manufacture. A stage-wise summary of parameters significantly affected by hurdle effects. **Bold:** multivariate findings; Underlined: recipe parameters; Non-bold: univariate findings by one-way ANOVA (The finding was independently significant, but not significant in the multivariate model, therefore provided as background exploratory information) (RLD).

Stage/Recipe Parameter	Significantly Affected Parameters
**Pre-Fermentation**	
Salt	Lactic acid (%) ↑, Salt (%) ↑, **TVC ↓, LAB ↓, Coliforms ↓.**
Nitrite	**Moisture ↓**, **Nitrate (ppm) ↑,** Nitrite (ppm) ↑, **TVC ↓, LAB ↓,** Coliforms ↓.
Salt × Nitrite	**TVC ↓, LAB ↓, Coliforms ↓.**
**Post-Fermentation**	
Salt	**Ash (%) ↑**, Salt (%) ↑, **LAB ↓, Coliforms ↓.**
Nitrite	**Moisture ↓**, Nitrite (ppm) ↑, TBARS ↑, ***Staphylococcus*** **spp.** ↓, Coliforms ↓, ETB ↓.
Salt × Nitrite	**TVC ↓, LAB ↓,** ***Staphylococcus*** **spp. ↓**
**Post-Heat Treatment**	
Salt	Lactic acid (%) ↓, **TVC ↓, LAB ↓.**
Nitrite	**TVC ↓, LAB ↓,** *Staphylococcus* spp. *↓*.
Heat	pH ^a^, **Lactic acid (%) ^b^, TVC ^c^**, LAB ^c^, ETB ^c^, *Pseudomonas* spp. ^d^, *B. thermosphacta* ^e^.
**Post-Drying**	
Salt	Moisture ↓, **Fat ↑**, Protein ↑, **Ash ↑**, aW ↑, pH ↑, **Lactic acid (%) ↓**, Salt (%) ↑, TBARS ↓, Hardness ↑, Cohesiveness ↑, Gumminess ↑, Resilience ↑, **TVC ↓, LAB ↓,** *Pseudomonas* spp. *↓*.
Nitrite	Moisture ↓, **Fat ↑**, Protein ↑, **Lactic acid (%) ↓**, **Nitrate (ppm) ↑**, Nitrite (ppm) ↑, L* ↑, a* ↑, b* ↑, **Cohesiveness ↑, TVC ↓, LAB ↓,** *B thermosphacta* ↓.
Heat	**Moisture ^f^, Fat ^f^**, Protein ^g^, Ash ^g^, aW ^f^, TBARS ^c^, L* ^g^, a* ^b^, b* ^g^, Adhesiveness ^d^, **TVC ^d^, LAB ^d^**, *Pseudomonas* spp. ^h^
aW	Moisture ↓, Fat ↑, Protein ↑, aW↓, Salt (%) ↑, TBARS ↓, Adhesiveness ↓, Cohesiveness ↑, Gumminess ↑, Chewiness ↑, Resilience ↑.
↑	The impact of hurdles resulted in an increase in the measured parameter following Benjamin-Hochberg FDR correction.
↓	The hurdles’ impact resulted in a decrease in the measured parameter following Benjamin-Hochberg FDR correction.
^a^	HT1 = HT2 > HT3
^b^	HT3 > HT1 > HT2
^c^	HT2 > HT1 > HT3
^d^	HT1 > HT2 > HT3
^e^	HT1 > HT2 = HT3
^f^	HT3 > HT2 > HT1
^g^	HT1 > HT3 > HT2
^h^	HT2 > HT1 = HT3

**Table 5:** Univariate effects following multivariate analysis (pre-fermentation). Univariate effects of recipe hurdles following multivariate analysis at the pre-fermentation stage. Panel A represents one- and two-way findings from the multivariate model. Panel B presents independent univariate findings via one-way ANOVA (RLD).

Panel A: Effects retained following multivariate modelling (primary)						
Recipe Parameter	Analyte	F	Sig.	Partial ε^2^	FDR q	FDR Sig.
Salt	Lactic acid (%)	7.4(1,94)	0.008	0.07	0.010	Yes
Salt	TVC	8.3(1,46)	0.006	0.16	0.025	Yes
Salt	LAB	4.5(1,46)	0.039	0.09	0.050	Yes
Salt	Coliforms	11.2(1,30)	0.005	0.44	0.008	Yes
Nitrite	Moisture	7.6(1,94)	0.007	0.08	0.013	Yes
Nitrite	Nitrate (ppm)	14.8(1,94)	0.000	0.14	0.010	Yes
Nitrite	TVC	5.6(1,46)	0.022	0.11	0.025	Yes
Salt × Nitrite	TVC	5.4(1,46)	0.025	0.11	0.050	Yes
Salt × Nitrite	LAB	76.5(1,46)	0.000	0.63	0.025	Yes
Salt × Nitrite	Coliforms	8.9(1,30)	0.010	0.39	0.017	Yes
Panel B: Exploratory one-way comparisons: one-way ANOVA (supporting)						
Recipe Parameter	Analyte	F	Sig.	Partial ε^2^	FDR q	FDR Sig.
Salt	Lactic acid (%)	7.3(1,94)	0.008	0.07	0.020	Yes
Salt	Salt (%)	194(1,94)	0.000	0.67	0.010	Yes
Salt	TVC	6.9(1,46)	0.012	0.13	0.025	Yes
Salt	Coliforms	17.5(1,30)	0.000	0.37	0.008	Yes
Nitrite	Moisture	7.3(1,94)	0.008	0.07	0.013	Yes
Nitrite	Nitrate (ppm)	15(1,94)	0.000	0.14	0.020	Yes
Nitrite	Nitrite (ppm)	2364(1,94)	0.000	0.96	0.010	Yes

**Table 6:** Univariate effects following multivariate analysis (post-fermentation). Univariate effects of recipe hurdles following multivariate analysis at the post-fermentation stage. Panel A represents one- and two-way findings from the multivariate model. Panel B presents independent univariate findings via one-way ANOVA (RLD).

Panel A: Effects retained following multivariate modelling (primary)						
Recipe Parameter	Analyte	F	Sig.	Partial ε^2^	FDR q	FDR Sig.
Salt	Ash	10.4(1,94)	0.002	0.10	0.013	Yes
Salt	LAB	9(1,46)	0.005	0.17	0.025	Yes
Salt	Coliforms	14(1,28)	0.004	0.58	0.008	Yes
Nitrite	Moisture	31.6(1,94)	0.000	0.26	0.013	Yes
Nitrite	*Staphylococcus* spp.	44.8(1,25)	0.000	0.82	0.008	Yes
Salt × Nitrite	Lactic acid (%)	8.9(1,94)	0.004	0.09	0.010	Yes
Salt × Nitrite	TVC	8.5(1,46)	0.006	0.16	0.050	Yes
Salt × Nitrite	LAB	46.8(1,46)	0.000	0.52	0.025	Yes
Salt × Nitrite	*Staphylococcus* spp.	32.2(1,25)	0.000	0.76	0.008	Yes
Panel B: Exploratory one-way ANOVA comparisons: (supporting)						
Recipe Parameter	Analyte	F	Sig.	Partial ε^2^	FDR q	FDR Sig.
Salt	Ash	10.4(1,94)	0.002	0.10	0.013	Yes
Salt	Salt (%)	311.9(1,94)	0.000	0.77	0.010	Yes
Salt	Coliforms	25(1,28)	0.000	0.47	0.008	Yes
Nitrite	Moisture	30.3(1,94)	0.000	0.24	0.013	Yes
Nitrite	Nitrite (ppm)	1858.5(1,94)	0.000	0.95	0.010	Yes
Nitrite	TBARS	6.3(1,94)	0.014	0.06	0.020	Yes
Nitrite	*Staphylococcus* spp.	12.9(1,25)	0.001	0.34	0.017	Yes
Nitrite	*Enterobacteriaceae*	14.1(1,30)	0.001	0.32	0.008	Yes

**Table 7:** Univariate effects following multivariate analysis (Post-Heat Treatment). Univariate effects of recipe hurdles following multivariate analysis at the post-heat treatment stage. Panel A represents one- and two-way findings from the multivariate model. Panel B presents independent univariate findings via one-way ANOVA (RLD) (Spoilage flora (BMD)).

Panel A: Effects retained following multivariate modelling (primary)						
Recipe Parameter	Analyte	F	Sig.	Partial ε^2^	FDR q	FDR Sig.
Salt	TVC	653.7(1,46)	<0.001	0.94	0.025	Yes
Salt	LAB	168.3(1,46)	<0.001	0.81	0.050	Yes
Nitrite	TVC	302.8(1,46)	<0.001	0.88	0.025	Yes
Nitrite	LAB	105.4(1,46)	<0.001	0.72	0.050	Yes
Heat Treatment	Lactic acid (%)	8.2(1,94)	<0.001	0.16	0.017	Yes
Heat Treatment	TVC	11.1(1,46)	<0.001	0.36	0.025	Yes
Panel B: Exploratory one-way comparisons: one-way ANOVA (supporting)						
Recipe Parameter	Analyte	F	Sig.	Partial ε^2^	FDR q	FDR Sig.
Salt	Lactic acid (%)	470.1(1,94)	<0.001	0.83	0.010	Yes
Salt	TVC	24.2(1,46)	<0.001	0.34	0.025	Yes
Salt	LAB	14.5(1,46)	<0.001	0.24	0.050	Yes
Salt	*Staphylococcus* spp.	4.3(1,20)	0.052	0.13	0.010	No
Salt	Coliforms	1.3(1,19)	0.272	0.01	0.020	No
Salt	*Enterobacteriaceae*	0.9(1,18)	0.350	0.00	0.030	No
Salt	*Pseudomonas* spp.	0.8(1,18)	0.390	−0.01	0.040	No
Salt	*B. thermosphacta*	0.1(1,19)	0.803	−0.05	0.050	No
Salt	Yeasts and Moulds	0(1,22)	-	-	-	-
Nitrite	TVC	4.7(1,46)	0.034	0.09	0.050	Yes
Nitrite	LAB	6.8(1,46)	0.012	0.13	0.025	Yes
Nitrite	*Staphylococcus* spp.	21.8(1,20)	<0.001	0.50	0.010	Yes
Nitrite	Coliforms	1.3(1,19)	0.272	0.01	0.020	No
Nitrite	*Enterobacteriaceae*	0(1,18)	0.886	−0.05	0.050	No
Nitrite	*Pseudomonas* spp.	0(1,18)	0.832	−0.05	0.040	No
Nitrite	*B. thermosphacta*	0.1(1,19)	0.803	−0.05	0.030	No
Nitrite	Yeasts and Moulds	0(1,22)	-	-		
Heat Treatment	pH	5.9(2,93)	0.004	0.11	0.010	Yes
Heat Treatment	Lactic acid (%)	4.2(2,93)	0.018	0.08	0.020	Yes
Heat Treatment	TVC	3.8(2,45)	0.029	0.15	0.050	Yes
Heat Treatment	LAB	10.3(2,45)	<0.001	0.31	0.025	Yes
Heat Treatment	*Staphylococcus* spp.	3.2(2,19)	0.066	0.17	0.040	No
Heat Treatment	Coliforms	3.1(2,18)	0.071	0.17	0.050	No
Heat Treatment	*Enterobacteriaceae*	111.3(2,17)	<0.001	0.92	0.010	Yes
Heat Treatment	*Pseudomonas* spp.	10.9(2,17)	0.001	0.51	0.020	Yes
Heat Treatment	*B. thermosphacta*	6.1(2,18)	0.010	0.34	0.030	Yes
Heat Treatment	Yeasts and Moulds	0(2,21)	-	-		

**Table 8:** Univariate effects following multivariate analysis (post-drying). Univariate effects of recipe hurdles following multivariate analysis at the post-drying stage. Panel A represents one- and two-way findings from the multivariate model. Panel B presents independent univariate findings via one-way ANOVA (RLD) (Spoilage flora (BMD)).

Panel A: Effects retained following multivariate modelling (primary)						
Recipe Parameter	Analyte	F	Sig.	Partial ε^2^	FDR q	FDR Sig.
Salt	Fat	8.1(1,94)	0.005	0.08	0.0250	Yes
Salt	Ash	17.7(1,94)	<0.001	0.17	0.0130	Yes
Salt	Lactic acid (%)	20.9(1,94)	<0.001	0.19	0.0100	Yes
Salt	TVC	115.7(2,45)	<0.001	0.74	0.0500	Yes
Salt	LAB	356.5(2,45)	<0.001	0.90	0.0250	Yes
Nitrite	Fat	22.1(1,94)	<0.001	0.20	0.0130	Yes
Nitrite	Lactic acid (%)	10.3(1,94)	0.002	0.11	0.0200	Yes
Nitrite	Nitrate (ppm)	23.8(1,94)	<0.001	0.21	0.0100	Yes
Nitrite	TBARS	9.4(2,93)	0.003	0.10	0.0300	Yes
Nitrite	a*	13.1(2,93)	<0.001	0.13	0.0170	Yes
Nitrite	Cohesiveness	17.2(1,94)	<0.001	0.16	0.0070	Yes
Nitrite	Resilience	16.2(1,94)	<0.001	0.16	0.0140	Yes
Nitrite	TVC	65.7(1,46)	<0.001	0.62	0.0500	Yes
Nitrite	LAB	240.8(1,46)	<0.001	0.86	0.0250	Yes
Heat Treatment	Moisture	10.3(1,94)	0.002	0.10	0.0250	Yes
Heat Treatment	Fat	19(1,94)	<0.001	0.18	0.0130	Yes
Heat Treatment	b*	14.5(2,93)	<0.001	0.14	0.0170	Yes
Heat Treatment	TVC	49.8(2,45)	<0.001	0.55	0.0250	Yes
Heat Treatment	LAB	14(2,45)	0.001	0.26	0.0500	Yes
Panel B: Exploratory one-way comparisons: one-way ANOVA (supporting)						
Recipe Parameter	Analyte	F	Sig.	Partial ε^2^	FDR q	FDR Sig.
Salt	Moisture	21.7(1,94)	<0.001	0.19	0.025	Yes
Salt	Fat	6.3(1,94)	0.014	0.06	0.050	Yes
Salt	Protein	8.1(1,94)	0.006	0.08	0.038	Yes
Salt	Ash	113.9(1,94)	<0.001	0.55	0.013	Yes
Salt	aW	8.3(1,94)	0.005	0.08	0.030	Yes
Salt	pH	7.2(1,94)	0.009	0.07	0.050	Yes
Salt	Lactic acid (%)	19.3(1,94)	<0.001	0.17	0.020	Yes
Salt	Salt (%)	446.7(1,94)	<0.001	0.83	0.010	Yes
Salt	TBARS	7.5(1,94)	0.007	0.07	0.040	Yes
Salt	Hardness	10.1(1,94)	0.002	0.10	0.021	Yes
Salt	Cohesiveness	5.6(1,94)	0.020	0.06	0.029	Yes
Salt	Gumminess	22.5(1,94)	<0.001	0.19	0.007	Yes
Salt	Resilience	12.4(1,94)	0.001	0.12	0.014	Yes
Salt	TVC	7.8(1,46)	0.008	0.14	0.050	Yes
Salt	LAB	9(1,46)	0.004	0.16	0.025	Yes
Salt	*Staphylococcus* spp.	0(1,22)	-	-		
Salt	Coliforms	2.2(1,22)	0.152	0.05	0.025	No
Salt	Enterobacteriaceae	1.1(1,20)	0.314	0.00	0.038	No
Salt	*Pseudomonas* spp.	11(1,22)	0.003	0.30	0.013	Yes
Salt	*B. thermosphacta*	0(1,21)	0.992	−0.05	0.050	No
Salt	Yeasts and Moulds	0(1,22)	-	-		
Nitrite	Moisture	26.4(1,94)	<0.001	0.22	0.013	Yes
Nitrite	Fat	23.8(1,94)	<0.001	0.20	0.025	Yes
Nitrite	Protein	10(1,94)	0.002	0.10	0.038	Yes
Nitrite	Nitrate (ppm)	59.6(1,94)	<0.001	0.39	0.020	Yes
Nitrite	Nitrite (ppm)	1238.3(1,94)	<0.001	0.93	0.010	Yes
Nitrite	L*	22.6(1,94)	<0.001	0.19	0.017	Yes
Nitrite	a*	21.2(1,94)	<0.001	0.18	0.033	Yes
Nitrite	b*	9.1(1,94)	0.003	0.09	0.050	Yes
Nitrite	Cohesiveness	9.7(1,94)	0.002	0.09	0.007	Yes
Nitrite	*Staphylococcus* spp.	0(1,22)	-	-	-	-
Nitrite	Coliforms	2.2(1,22)	0.152	0.05	0.025	No
Nitrite	Enterobacteriaceae	0(1,20)	0.961	−0.05	0.038	No
Nitrite	*Pseudomonas* spp.	0(1,22)	0.973	−0.05	0.050	No
Nitrite	*B. thermosphacta*	8.7(1,21)	0.008	0.26	0.013	Yes
Nitrite	Yeasts and Moulds	0(1,22)	-	-	-	-
Heat Treatment	Moisture	18.8(2,93)	<0.001	0.29	0.025	Yes
Heat Treatment	Fat	18.6(2,93)	<0.001	0.29	0.038	Yes
Heat Treatment	Protein	28.4(2,93)	<0.001	0.38	0.013	Yes
Heat Treatment	Ash	3.1(2,93)	0.048	0.06	0.050	Yes
Heat Treatment	aW	18.8(2,93)	<0.001	0.29	0.020	Yes
Heat Treatment	TBARS	43.6(2,93)	<0.001	0.48	0.010	Yes
Heat Treatment	L*	16.5(2,93)	<0.001	0.26	0.033	Yes
Heat Treatment	a*	13.8(2,93)	<0.001	0.23	0.050	Yes
Heat Treatment	b*	36.1(2,93)	<0.001	0.44	0.017	Yes
Heat Treatment	Adhesiveness	6.4(2,93)	0.002	0.12	0.007	Yes
Heat Treatment	TVC	17.5(2,45)	<0.001	0.44	0.025	Yes
Heat Treatment	LAB	9.8(2,45)	<0.001	0.30	0.050	Yes
Heat Treatment	*Staphylococcus* spp.	0(2,21)	-	-		
Heat Treatment	Coliforms	1.9(2,21)	0.178	0.07	0.038	No
Heat Treatment	*Enterobacteriaceae*	3.4(2,19)	0.055	0.18	0.025	No
Heat Treatment	*Pseudomonas* spp.	13.1(2,21)	<0.001	0.51	0.013	Yes
Heat Treatment	*B. thermosphacta*	1(2,20)	0.369	0.00	0.050	No
Heat Treatment	Yeasts and Moulds	0(2,21)	-	-		
aW	Moisture	16.4(1,94)	<0.001	0.15	0.038	Yes
aW	Fat	16.7(1,94)	<0.001	0.15	0.025	Yes
aW	Protein	37.3(1,94)	<0.001	0.28	0.013	Yes
aW	aW	252(1,94)	<0.001	0.73	0.010	Yes
aW	Salt (%)	5.4(1,94)	0.022	0.05	0.030	Yes
aW	TBARS	11.1(1,94)	0.001	0.11	0.020	Yes
aW	Adhesiveness	20(1,94)	<0.001	0.18	0.007	Yes
aW	Cohesiveness	6.1(1,94)	0.016	0.06	0.029	Yes
aW	Gumminess	11.9(1,94)	0.001	0.11	0.021	Yes
aW	Chewiness	13.3(1,94)	<0.001	0.12	0.014	Yes
aW	Resilience	5.2(1,94)	0.025	0.05	0.036	Yes
aW	*Staphylococcus* spp.	0(1,22)	-	-		
aW	Coliforms	0(1,22)	0.060	0.11	0.025	No
aW	*Enterobacteriaceae*	0(1,22)	0.590	−0.03	0.050	No
aW	*Pseudomonas* spp.	0(1,22)	0.465	−0.02	0.038	No
aW	*B. thermosphacta*	0(1,22)	0.049	0.13	0.013	No
aW	Yeasts and Moulds	0(1,22)	-	-		

**Table 9:** Microbiology of the Food Chain—Preparation of Test SamDescriptive trends for parameters significantly affected by salt. Stage-wise descriptive trends (mean ± se) of parameters significantly affected by salt in univariate and multivariate analyses. Shaded cells indicate significant effects following FDR correction (RLD).

Analyte	Salt (%)	Pre	Fermented	Heat	Dry
Moisture (%)	1.4	50.36 ± 0.17	49.02 ± 0.41	51.12 ± 0.2	40.09 ± 0.2
	2.5	49.78 ± 0.22	48.78 ± 0.2	51.24 ± 0.18	38.63 ± 0.24
Fat (%)	1.4	23.54 ± 0.27	23.04 ± 0.21	21.01 ± 0.29	27.25 ± 0.17
	2.5	23.4 ± 0.24	23.27 ± 0.24	20.38 ± 0.25	27.99 ± 0.24
Protein (%)	1.4	12.71 ± 0.18	13.19 ± 0.31	-	17.02 ± 0.25
	2.5	13.23 ± 0.15	12.96 ± 0.18	-	18.06 ± 0.26
Ash (%)	1.4	2.9 ± 0.04	2.95 ± 0.04	2.9 ± 0.05	4.15 ± 0.07
	2.5	3.77 ± 0.62	3.15 ± 0.04	3.01 ± 0.04	5.05 ± 0.05
aW	1.4	0.98 ± 0	0.98 ± 0	1 ± 0	0.92 ± 0
	2.5	0.99 ± 0	0.98 ± 0	1 ± 0	0.91 ± 0
pH	1.4	5.57 ± 0.01	5.05 ± 0.01	5.03 ± 0.01	4.67 ± 0.01
	2.5	5.6 ± 0.02	5.04 ± 0.01	5.03 ± 0.01	4.71 ± 0.01
Lactic acid (%)	1.4	0.41 ± 0.01	0.64 ± 0.01	0.63 ± 0.01	0.93 ± 0.01
	2.5	0.44 ± 0.01	0.63 ± 0.01	0.59 ± 0.01	0.85 ± 0.01
Salt (%)	1.4	1.73 ± 0.05	1.81 ± 0.03	1.69 ± 0.03	2.83 ± 0.03
	2.5	2.59 ± 0.04	2.68 ± 0.04	2.48 ± 0.02	3.86 ± 0.04
TBARS (mgMDA/kg)	1.4	4.63 ± 0.12	6.96 ± 0.22		8.63 ± 0.28
	2.5	4.42 ± 0.09	6.31 ± 0.21		7.57 ± 0.27
Hardness	1.4				685.23 ± 11.44
	2.5				732.86 ± 9.7
Cohesiveness	1.4				0.52 ± 0.01
	2.5				0.5 ± 0.01
Gumminess	1.4				335.75 ± 7.93
	2.5				396.39 ± 10.01
Resilience	1.4				0.13 ± 0
	2.5				0.12 ± 0
TVC	1.4	8.5 ± 0.12	8.79 ± 0.12	8.71 ± 0.09	9.16 ± 0.15
	2.5	7.96 ± 0.17	8.39 ± 0.18	7.47 ± 0.24	8.49 ± 0.18
LAB	1.4	8.5 ± 0.12	8.79 ± 0.12	8.71 ± 0.09	9.16 ± 0.15
	2.5	8.27 ± 0.13	8.35 ± 0.16	7.86 ± 0.21	8.42 ± 0.19
*Staphylococcus* spp.	1.4	3.13 ± 0.11	3.1 ± 0.04	2.65 ± 0.15	-
	2.5	3.12 ± 0.17	3.08 ± 0.27	0.97 ± 0.39	-
Coliforms	1.4	4.17 ± 0.27	3.88 ± 0.13	0.82 ± 0.37	0.25 ± 0.17
	2.5	2.1 ± 0.39	1.78 ± 0.4	0.32 ± 0.22	0 ± 0
*Enterobacteriaceae*	1.4	4.02 ± 0.18	2.47 ± 0.44	2.12 ± 0.44	0.86 ± 0.36
	2.5	3.89 ± 0.24	3.02 ± 0.13	1.36 ± 0.47	0.51 ± 0.24
*Pseudomonas* spp.	1.4	4.56 ± 0.17	3.42 ± 0.16	1.99 ± 0.42	1.34 ± 0.42
	2.5	4.23 ± 0.24	3.11 ± 0.33	0.9 ± 0.4	0 ± 0
*B. thermosphacta*	1.4	4.47 ± 0.24	3.62 ± 0.27	0.22 ± 0.16	0.39 ± 0.18
	2.5	4.65 ± 0.3	3.63 ± 0.34	0.16 ± 0.16	0.3 ± 0.21
Yeasts and Moulds	1.4	2.63 ± 0.57	2.13 ± 0.52	0 ± 0	-
	2.5	2.17 ± 0.48	1.82 ± 0.42	0 ± 0	-

**Table 10:** Descriptive trends for parameters significantly affected by nitrite. Stage-wise descriptive trends (mean ± se) of parameters significantly affected by nitrite concentration in univariate and multivariate analyses. Shaded cells indicate significant effects following FDR correction (RLD) (Spoilage flora (BMD)).

	Nitrite (ppm)	Pre	Fermented	Heat	Dry
Moisture (%)	50 ppm	50.44 ± 0.2	49.99 ± 0.22	51.29 ± 0.19	40.15 ± 0.19
	150 ppm	49.69 ± 0.18	47.82 ± 0.33	51.07 ± 0.19	38.57 ± 0.24
Fat (%)	50 ppm	23.3 ± 0.26	23.27 ± 0.16	20.78 ± 0.26	26.96 ± 0.17
	150 ppm	23.64 ± 0.24	23.05 ± 0.28	20.61 ± 0.29	28.28 ± 0.21
Protein(%)	50 ppm	13.09 ± 0.18	13.16 ± 0.14	-	16.97 ± 0.27
	150 ppm	12.85 ± 0.16	12.99 ± 0.33	-	18.11 ± 0.24
Lactic acid (%)	50 ppm	0.43 ± 0.01	0.63 ± 0.01	0.61 ± 0.01	0.9 ± 0.01
	150 ppm	0.42 ± 0.01	0.64 ± 0.01	0.61 ± 0.01	0.87 ± 0.01
Nitrate (ppm)	50 ppm	11.64 ± 0.27	15.88 ± 2.12	-	16.37 ± 0.25
	150 ppm	12.99 ± 0.21	16.61 ± 2.08	-	19.45 ± 0.31
Nitrite (ppm)	50 ppm	57.71 ± 2.58	76.75 ± 2.64	-	126.96 ± 3.32
	150 ppm	155.46 ± 3.14	173.58 ± 3.73	-	246.23 ± 5.28
TBARS (mgMDA/kg)	50 ppm	4.43 ± 0.11	6.26 ± 0.2	-	7.84 ± 0.31
	150 ppm	4.62 ± 0.11	7.01 ± 0.22	-	8.37 ± 0.25
L*	50 ppm	-	-	-	61.29 ± 0.46
	150 ppm	-	-	-	63.83 ± 0.27
a*	50 ppm	-	-	-	12.58 ± 0.22
	150 ppm	-	-	-	13.8 ± 0.15
b*	50 ppm	-	-	-	21.29 ± 0.38
	150 ppm	-	-	-	22.66 ± 0.24
Cohesiveness	50 ppm	-	-	-	0.49 ± 0.01
	150 ppm	-	-	-	0.52 ± 0.01
TVC	50 ppm	8.01 ± 0.18	8.44 ± 0.17	8.42 ± 0.11	8.85 ± 0.22
	150 ppm	8.46 ± 0.12	8.74 ± 0.14	7.77 ± 0.28	8.8 ± 0.12
LAB	50 ppm	8.42 ± 0.11	8.62 ± 0.14	8.6 ± 0.09	8.89 ± 0.12
	150 ppm	8.36 ± 0.14	8.52 ± 0.16	7.97 ± 0.22	8.69 ± 0.24
*Staphylococcus* spp.	50 ppm	3.17 ± 0.15	3.36 ± 0.11	3.1 ± 0.16	0 ± 0
	150 ppm	3.08 ± 0.12	2.64 ± 0.18	1.1 ± 0.39	0 ± 0
Coliforms	50 ppm	3.73 ± 0.22	3.51 ± 0.23	1.3 ± 0.54	0.5 ± 0.34
	150 ppm	2.22 ± 0.57	2.37 ± 0.42	0.6 ± 0.39	0 ± 0
*Enterobacteriaceae*	50 ppm	3.99 ± 0.24	3.57 ± 0.16	1.89 ± 0.47	0.8 ± 0.28
	150 ppm	3.91 ± 0.18	1.95 ± 0.38	1.72 ± 0.47	0.56 ± 0.31
*Pseudomonas* spp.	50 ppm	4.69 ± 0.16	3.52 ± 0.2	1.56 ± 0.41	0.67 ± 0.31
	150 ppm	4.1 ± 0.24	3.05 ± 0.3	1.21 ± 0.43	0.52 ± 0.28
*B. thermosphacta*	50 ppm	4.7 ± 0.28	3.88 ± 0.36	0.22 ± 0.16	0.81 ± 0.28
	150 ppm	4.41 ± 0.27	3.49 ± 0.25	0.16 ± 0.16	0 ± 0
Yeasts and Moulds	50 ppm	3.02 ± 0.51	2.48 ± 0.46	0 ± 0	-
	150 ppm	1.73 ± 0.49	1.44 ± 0.43	0 ± 0	-

**Table 11:** Descriptive trends for parameters significantly affected by heat treatment. Descriptive trends (mean ± se) of parameters significantly affected by heat treatment in multivariate and univariate analysis. Shaded cells indicate parameters which were significantly impacted following FDR correction (RLD) (Spoilage flora (BMD)).

	Recipe Factor	Heat	Dry
Moisture (%)	53 °C 60 min	50.73 ± 0.21	38.62 ± 0.16
	61 °C 40 min	51.31 ± 0.22	40.53 ± 0.17
	64 °C 20 min	51.66 ± 0.25	38.73 ± 0.48
Fat (%)	53 °C 60 min	20.88 ± 0.32	28.61 ± 0.22
	61 °C 40 min	20.45 ± 0.33	26.87 ± 0.2
	64 °C 20 min	20.78 ± 0.39	27.25 ± 0.25
Protein (%)	53 °C 60 min		18.94 ± 0.27
	61 °C 40 min		16.36 ± 0.2
	64 °C 20 min		17.2 ± 0.32
Ash (%)	53 °C 60 min	3.02 ± 0.05	4.79 ± 0.09
	61 °C 40 min	2.89 ± 0.06	4.44 ± 0.11
	64 °C 20 min	2.95 ± 0.04	4.54 ± 0.13
aW	53 °C 60 min	1 ± 0	0.9 ± 0
	61 °C 40 min	1 ± 0	0.93 ± 0
	64 °C 20 min	1 ± 0	0.92 ± 0
pH	53 °C 60 min	5.04 ± 0.01	4.69 ± 0.01
	61 °C 40 min	5.04 ± 0.01	4.69 ± 0.01
	64 °C 20 min	5.02 ± 0.01	4.69 ± 0.02
Lactic acid (%)	53 °C 60 min	0.6 ± 0.01	0.89 ± 0.02
	61 °C 40 min	0.59 ± 0.01	0.88 ± 0.02
	64 °C 20 min	0.65 ± 0.01	0.9 ± 0.02
TBARS (mgMDA/kg)	53 °C 60 min		7.26 ± 0.18
	61 °C 40 min		9.83 ± 0.33
	64 °C 20 min		6.77 ± 0.14
L*	53 °C 60 min		63.88 ± 0.22
	61 °C 40 min		60.66 ± 0.56
	64 °C 20 min		63.41 ± 0.49
a*	53 °C 60 min		13.27 ± 0.19
	61 °C 40 min		12.44 ± 0.25
	64 °C 20 min		14.19 ± 0.21
b*	53 °C 60 min		23.44 ± 0.2
	61 °C 40 min		20.06 ± 0.33
	64 °C 20 min		22.66 ± 0.42
Adhesiveness	53 °C 60 min		−59.44 ± 4.89
	61 °C 40 min		−88.32 ± 11.67
	64 °C 20 min		−105.99 ± 8.59
TVC	53 °C 60 min	7.97 ± 0.34	9.44 ± 0.18
	61 °C 40 min	8.57 ± 0.15	8.79 ± 0.14
	64 °C 20 min	7.56 ± 0.18	7.95 ± 0.18
LAB	53 °C 60 min	8.24 ± 0.25	9.23 ± 0.25
	61 °C 40 min	8.82 ± 0.09	8.91 ± 0.12
	64 °C 20 min	7.56 ± 0.18	7.95 ± 0.18
*Staphylococcus* spp.	53 °C 60 min	1.16 ± 0.42	-
	61 °C 40 min	2.98 ± 0.25	-
	64 °C 20 min	1.01 ± 0.51	-
Coliforms	53 °C 60 min	0.46 ± 0.31	0 ± 0
	61 °C 40 min	1.31 ± 0.54	0.34 ± 0.23
	64 °C 20 min	0 ± 0	0 ± 0
*Enterobacteriaceae*	53 °C 60 min	3.16 ± 0.05	0.69 ± 0.31
	61 °C 40 min	3.37 ± 0.28	1.23 ± 0.49
	64 °C 20 min	0 ± 0	0 ± 0
*Pseudomonas* spp.	53 °C 60 min	3.09 ± 0.24	0 ± 0
	61 °C 40 min	1.89 ± 0.51	1.96 ± 0.53
	64 °C 20 min	0 ± 0	0 ± 0
*B. thermosphacta*	53 °C 60 min	0.84 ± 0.43	0.43 ± 0.29
	61 °C 40 min	0 ± 0	0.54 ± 0.24
	64 °C 20 min	0 ± 0	0 ± 0
Yeasts and Moulds	53 °C 60 min	0 ± 0	-
	61 °C 40 min	0 ± 0	-
	64 °C 20 min	0 ± 0	-

**Table 12:** Descriptive trends for parameters significantly affected by aW. Descriptive trends (mean ± se) of parameters significantly affected by aW level in univariate analysis. Shaded cells indicate parameters which were significantly impacted by aW level following FDR correction (RLD).

	Recipe Factor	Dry
Moisture (%)	0.91	38.86 ± 0.24
	0.94	40.2 ± 0.15
Fat (%)	0.91	28.06 ± 0.19
	0.94	26.88 ± 0.18
Protein (%)	0.91	18.3 ± 0.22
	0.94	16.27 ± 0.21
aW	0.91	0.9 ± 0
	0.94	0.94 ± 0
Salt (%)	0.91	3.45 ± 0.07
	0.94	3.18 ± 0.1
TBARS (mgMDA/kg)	0.91	7.61 ± 0.18
	0.94	8.92 ± 0.41
Adhesiveness	0.91	−64.5 ± 4.99
	0.94	−110.93 ± 10.52
Cohesiveness	0.91	0.52 ± 0
	0.94	0.5 ± 0.01
Gumminess	0.91	395.94 ± 12.63
	0.94	348.15 ± 7.59
Chewiness	0.91	270.11 ± 5.4
	0.94	241.38 ± 5.18
Resilience	0.91	0.13 ± 0
	0.94	0.12 ± 0

## Data Availability

The original contributions presented in the study are included in the article/Supplementary Materials, further inquiries can be directed to the corresponding author

## Supplementary Materials

The following supporting information can be downloaded at: https://www.mdpi.com/article/10.3390/foods15111952/s1.
